# Variations in Leaf Traits Modulate Plant Vegetative and Reproductive Phenological Sequencing Across Arid Mediterranean Shrublands

**DOI:** 10.3389/fpls.2021.708367

**Published:** 2021-08-23

**Authors:** Teresa Navarro, Noelia Hidalgo-Triana

**Affiliations:** Department of Botany and Plant Physiology (Botany Area), Faculty of Science, University of Malaga, Malaga, Spain

**Keywords:** arid Mediterranean climate, leaf economics spectrum, nitrogen, phosphorus, perennial species, phenophase sequence index, specific leaf area, stoichiometry

## Abstract

Structural and nutrient traits of a leaf are important for understanding plant ecological strategies (e.g., drought avoidance). We studied the specific leaf area (SLA), leaf carbon content (LCC), leaf nitrogen content (LNC), leaf phosphorous content (LPC), and the phenophase sequence index (PSI) in 126 Mediterranean perennial species from predesert (SMS) and semiarid (SaMS) to subalpine (SAS), alpine cushion (AcS), and oro-Mediterranean (AjS) shrublands, which represent eight functional groups (evergreen and deciduous trees, evergreen large and half shrubs, deciduous large and half shrubs, succulents and perennial herbs). We analyzed the variation and relationships between leaf traits and PSI among shrublands, functional groups, and within species with drought-avoidance mechanisms. SLA variation of 20–60% could be ascribed to differences between functional groups and only 38–48% to different shrublands increasing from the predesert to the alpine. Alpine species display low PSI and N:P and high SLA, LNC, LPC, LCC, and C:N. On the contrary, predesert and semiarid showed high PSI and low SLA. SLA mediates the vegetative and reproductive phenological plant sequencing, high SLA is often associated with the overlapping in growth and reproductive phenophases with a seasonal reduction of vegetative growth, whereas low SLA is associated with vegetative and reproductive sequencing and a seasonal extension of vegetative growth. Species with drought-avoidance mechanisms (e.g., semideciduous species) contribute to an increase in the mean values of the SLA and LNC because these species show similar leaf and phenological patterns as the deciduous (high SLA and LNC and low PSI). The N:P indicates that only the alpine shrublands could present P limitations. The positive correlations between SLA and LPC and LNC and LPC (leaf economic spectrum) and the negative correlation between SLA and C:N were consistently maintained in the studied arid Mediterranean shrublands.

## Introduction

Exploring structural and nutrient leaf traits in arid Mediterranean shrublands provides new insights into ecosystem functioning, services, and vulnerability (Díaz et al., 2004; He et al., 2006). Although changes in vegetative and reproductive phenology are the most evident plant responses to climate, it is not known whether they are mediated by leaf traits in drylands (Valencia et al., 2016). Specific leaf area (SLA), leaf nitrogen content (LNC), and leaf phosphorus content (LPC) are part of the leaf economic spectrum (Wright et al., 2004, 2007). The SLA is related to the relative growth rate (RGR) (Reich et al., 1997), which has important ecological consequences in plant seasonal vegetative renewal (Castro-Díez et al., 2003). High values of SLA, LNC, and RGR (Reich et al., 1998) are associated with a reduced vegetative growth phase and high potential in terms of carbon acquisition (Díaz et al., 2004). Such species are associated with the lowest drought survival time (Lopez-Iglesias et al., 2014). This set of traits has been described as acquisitive traits (Díaz et al., 2004). Species with low SLA, LNC, and RGR values are associated with a low potential for carbon acquisition (Díaz et al., 2004). Plants vary in their phenological behavior according to morphological traits related to resource conservation and acquisition (Castro-Díez et al., 2003). Campanella and Bertiller (2008) revealed that the maintenance of the vegetative growth phase during the dry season is mostly associated with leaves possessing low SLA.

The responses of SLA, LNC, and LPC to climate are associated with variations in functional groups (He et al., 2006), which is crucial for predicting the loss of biodiversity (Wright et al., 2005). The general trend shows that deciduous species and herbs present higher values of SLA, LNC, and LPC than evergreens (Reich et al., 1998). However, this general trend has been poorly studied in semideciduous species that are linked to more xeric conditions in Mediterranean ecosystems (Travlos and Chachalis, 2008), with photosynthetic organs ready to work when the climatic conditions become most favorable (Correia and Ascensão, 2017).

Leaf stoichiometry, such as the N:P ratio, is important for the modeling and recycling of carbon (Sterner and Elser, 2002) and the functioning of ecosystems and their management (Wright et al., 2004). The N:P ratio influences vegetative growth and the reproduction of individual plants (Güsewell, 2004). However, the models of N and P limitation in relation to the foliar LNC and LPC are not well characterized (Elser et al., 2007). The N:P relationship is used to determine whether N or P is the more important limiting factor for biomass production. Variations in these limiting factors cause changes in functional characteristics, vegetation composition, and species diversity (Niklas and Cobb, 2005). For example, the limitation of P facilitates the coexistence of species where interspecific competition is greater. Thus, the N:P balance is of particular interest while studying the allocation of resources in species of ecosystems with summer drought, as the availability of N and/or P concentrations could limit plant growth (Güsewell, 2004). Some studies suggest that warming can lead to increases in N:P ratios in plants; however, more studies are necessary to gain more insight (Sardans and Peñuelas, 2012).

Plants frequently present higher C-nutrient ratios under drought conditions. It is of interest to investigate the change in C:N, particularly in Mediterranean plants which have a great capacity of resource remobilization throughout the year (Sardans et al., 2008), similar to the sequential phenological species.

Mediterranean woody species have developed structural, morphological, and physiological leaf traits that allow them to survive under summer drought stress (Harley et al., 1987). Deciduous and semideciduous species are frequent in the Mediterranean (Montserrat-Martí et al., 2004; Navarro et al., 2006, 2009a,b; Puglielli, 2019), and they rely on leaf phenology and structural and/or functional features to overcome summer drought stress, which is the primary constraint to the productivity and dynamics of the Mediterranean vegetation. In deciduous and semideciduous species, vegetative growth and reproduction are synchronized in spring and/or summer (Montserrat-Martí et al., 2004). The estimation of the degree of duration and the overlapping between vegetative and reproductive phenophases (phenophase sequence index (PSI)) (Castro Díez and Montserrat-Martí, 1998) are decisive features determining plant adaptation and persistence in seasonal environments (Castro Díez and Montserrat-Martí, 1998), which are associated with summer drought (Roche et al., 1997) and climate unpredictability (Correia and Ascensão, 2017).

Although Mediterranean ecosystems provide a good setting for examining functional traits (Ackerly et al., 2002), only a few reports have been published on leaf-trait covariation in perennial plants associated with functional groups. Seasonal dimorphism has long been considered a key adaptation to Mediterranean climate seasonality, including the oro-Mediterranean mountain (Niinemets, 2001; Navarro et al., 2009a,b). Dimorphism in semideciduous species is a strategy to avoid summer drought and winter cold in the Mediterranean climate (Palacio and Montserrat-Martí, 2006; Puglielli and Varone, 2018). These species reduce their green structures during summer to brachyblasts (Orshan, 1964; Dominguez et al., 2012; Puglielli, 2019).

The objective of this study was to explore the (co)variation patterns of leaf structural traits (SLA), leaf nutrient traits [leaf carbon content (LCC), leaf nitrogen content (LNC), and leaf phosphorous content (LPC)], stoichiometry (C:N:P), and PSI phenological traits in 126 perennial species distributed across five shrublands in southeast Spain covering desert, semiarid, and alpine Mediterranean climates. These shrublands cover a wide sample of the main and most climatically vulnerable shrubland types in the East of the Mediterranean basin (Sardans and Peñuelas, 2013).

We explored the variation and relationships at the species, Mediterranean arid shrublands and plant functional group levels, and we hypothesized the following: (1) SLA and leaf nutrients mediate in the vegetative and reproductive phenological plant sequencing through the reduction of vegetative growth phase; (2) species with drought-avoidance mechanisms contribute to an increase in the mean values of the SLA and LNC mainly due to the effect of semideciduous species which, shows similar patterns to deciduous (high SLA and LNC and low PSI); (3) the strong relationships among leaf functional traits and leaf economics spectrum is maintained in arid Mediterranean shrublands through the strong covariation between SLA and leaf nutrients in fast-growing species. Finally, we compared the variation in leaf functional trait values from Mediterranean shrublands with other world and Mediterranean ecosystems previously reported (Villar and Merino, 2001; Güsewell, 2004; Peter and Oleksyn, 2004; Wright et al., 2004; Villar et al., 2006; Fyllas et al., 2009, 2020; Hernández et al., 2011; Dominguez et al., 2012; De la Riva et al., 2016, 2017). Overall, this study serves as a relevant plant trait database for functional ecology in the Mediterranean shrublands affected by drought (Tavşanoglu and Pausas, 2018).

## Materials and Methods

### Study Area

**Figure 1:**
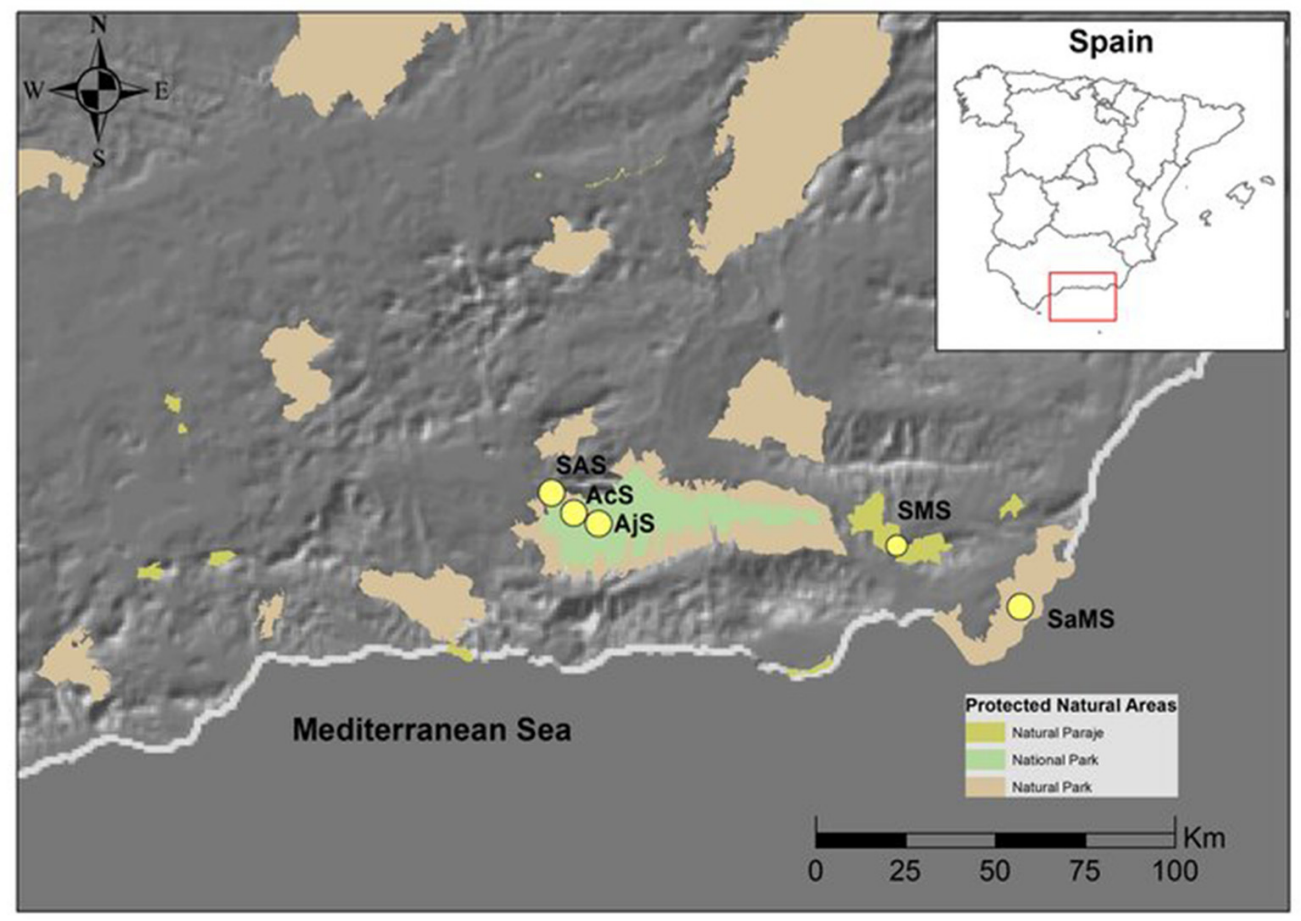
Location of the study area in the Iberian Peninsula (Andalusia, Spain). The yellow circles indicate the study areas in the natural protected areas. Modified from DERA. SaMS, semiarid mediterranean shrubland; SMS, subdesert mediterranean shrubland; SAS, subalpine shrubland; AcS, alpine cushion shrubland; and AjS, alpine juniper prostrate shrubland.

This study was conducted in the southeast of the Iberian Peninsula (Figure 1). The climate is of an arid Mediterranean type III (Martonne, 1926) with a dry summer season extending from June to the end of August and a rainy season from September to the end of February (April in the Sierra Nevada Mountains). The study was conducted in five Mediterranean shrublands located in the aridest region of the Iberian Peninsula (De Castro et al., 2005; Figure 1). The five sites were selected based on their rainfall and altitude diversity to sample a crosssection of arid, sub-desert, and mountain Mediterranean vegetation following Loidi (2017).

The shrublands were classified following the European classification of natural habitats (European Commission 2013). The soil type was realized according to the European Classification of Soils (2008). The bioclimatic classification was performed according to the method described by Rivas-Martínez (1996-2009). The study area was comprised of the following: (1) thermo-Mediterranean and predesert scrub (Cortijo Montano; Cabo de Gata-Nijar, Natural Park) (36°51′34.95″N 2° 4′41.38″O) a semiarid Mediterranean shrubland (SaMS), 132 m a.s.l., Calcaric Regosols, mean annual rainfall of 268 mm; (2) Mediterranean predesert scrub (Tabernas Paraje Natural) (37° 1′23.67″N 2°25′45.66″O) a subdesert Mediterranean shrubland (SMS), 343 m a.s.l., Calcaric Xerosols, mean annual rainfall of 221 mm; (3) stable xerothermophilous shrubland (Barranco de las Viboras; Sierra Nevada Natural Park) (37° 7′57.41″N 3°26′47.25″O) a subalpine shrubland (SAS), 1,419 m a.s.l., Cromis Luvisols, mean annual rainfall of 740 mm; (4) stable xerothermophilous shrubland (Collado de las Sabinas; Sierra Nevada Natural Park) (37° 6′57.59″N 3°25′20.87″O) an alpine cushion shrubland (AcS), 2,200 m a.s.l., Eutric Cambisols, mean annual rainfall of 730 mm; and (5) endemic oro-Mediterranean heaths (Mojon del Trigo; Sierra Nevada Natural Park) (37° 5′29.43″N 3°22′53.98″O), an alpine Juniper prostrate shrubland (AjS) 2,463 m a.s.l., Dystric Regosols, mean annual rainfall of 289 mm. The three last scrublands are located in the Sierra Nevada mountain (Figure 1).

### Species Selection

Woody plants and perennial herbaceous species were selected (see Supplementary Material 1: Table S1) based on the following criteria: (1) the species provide a continuous range of leaf traits based on prior ecological and floristic knowledge and (2) have the highest cover values based on previous studies (Navarro et al., 2006, 2010; Alados et al., 2017).

A total of 126 species belonging to 26 different taxonomic families and 74 genera were analyzed as highly representative of the vegetation diversity in the study area (Blanca et al., 2011). Voucher specimens of the studied species were stored in the MGC Herbarium. Botanical nomenclature followed POWO. (2019) and Castroviejo (1986–2019). A total of 1,372 data points were obtained and included in this dataset.

### Data Collection

Field sampling and phenological observations (vegetative and reproductive phenophases) were carried out monthly (December 2011–November 2014) following the method of Castro Díez and Montserrat-Martí (1998), where a phenophase is considered to be active in the population when it was observed in at least 5% of the crown of the plant and a minimum of 20% of the studied individuals up to a minimum of 10 individual plants per each species. Phenological seasonal calendars were averaged for each species (see Supplementary Material 2), which synthetically show the frequency of the occurrence of vegetative and reproductive phenophases. To estimate the degree of vegetative and reproductive phenophase sequencing, the PSI was calculated:

(1) $$\begin{array}{r} PSI=\frac{t\left[DVG+FBF+F\right]}{\left[t\left(DVG\right)+t\left(FBF\right)+t\left(F\right)\right]} \end{array}$$

where *t* is the number of months needed to complete the phenophase(s) indicated between parentheses, and the studied phenophases were the following: DVG, dolichoblast vegetative growth; FBF, flower bud formation; F, flowering (Castro Díez and Montserrat-Martí, 1998), which varies from 0.33 to 1. High index values indicate a sequential organization (PSI ≥ 0.6; sequencing species), whereas low values are related to a greater degree of overlap (PSI < 0.6; overlapping species).

First, we classified each species in functional groups following Pérez-Harguindeguy et al. (2013). Based on the leaf phenology (Tavşanoglu and Pausas, 2018), we differentiated between deciduous and evergreen species. Second, we classified the species into eight functional groups as per Poorter et al. (2009), namely, evergreen trees (ETs), deciduous trees (DTs), evergreen large shrubs (ELSs), evergreen half shrubs (EHSs), deciduous large shrubs (DLSs), deciduous half shrubs (DHSs), succulents (SCs), and perennial herbs (PHs). Only four species were sclerophyllous (*Juniperus sabina, Quercus coccifera, Q. ilex* subsp. *ballota, Olea europaea* var. *sylvestris*, and *Chamaerops humilis*; refer to Supplementary Material 1: Table S1). The semideciduous species with seasonal dimorphism [plants that reduced their dolichoblast leaves during summer to brachyblasts following Orshan (1964)] and species with green stems and ephemeral leaves were included into the EHSs. The analysis of leaf structure and nutrient content traits was conducted following the criteria defined by Pérez-Harguindeguy et al. (2013). Fully expanded and hardened leaves were collected throughout the year (mainly in spring) from random adult plants in full-light situations and without obvious symptoms of the pathogen, herbivore attack, or substantial cover of epiphylls (on robust and well-grown individual plants according to Cornelissen et al., 2003). Twenty leaves were collected from at least 10 individuals of each species. Leaves were transported to the laboratory in plastic bags and stored at low temperatures (2–6°C) for less than 24 h prior to measurements. SLA is the one-sided area of a fresh leaf divided by its oven-dry mass (Pérez-Harguindeguy et al., 2013). Leaf dry mass was determined after oven drying at 60°C for at least 72 h. Leaf area measurements of each individual leaf were estimated using Visilog 6.0 image analysis software (Noesis, Courtaboeuf, France).

For leaf nutrient content (LCC, LNC, and LPC), additional leaves were collected from each individual for chemical analysis. The leaves were ground using a mortar and pestle. The LCC and LNC were obtained using an elemental analyzer (Perkin-Elmer 2400 Series II, USA) whereas LPC was determined *via* dissolution in concentrated HCl and subsequent analysis with 5800 ICP OES, USA) (Varian Vista MPX).

In addition, drought-avoidance leaf traits (as the major limitation to the productivity of plants) were identified following the method of Travlos and Chachalis (2008). Seasonal dimorphism with leaf rolling during stress (White et al., 1992), leaves containing more trichomes or leaf indument (De Micco and Aronne, 2012; Sardans and Peñuelas, 2013), and plants with photosynthetic stems or green leafy shoots (stem-like leaf; Travlos and Chachalis, 2008) were assessed (refer to Supplementary Material 1: Table S1).

### Statistical Analysis

All statistical analyses were performed using SPSS (version 18.0, SPSS Inc., USA). All variables were log_10_-transformed prior to the analysis to normalize their distributions. The Kolmogorov–Smirnov test confirmed the normality assumption (*p* < 0.05). The transformed data were analyzed at two levels: (1) all of the species (full data set) and (2) averaging across Mediterranean shrublands and functional groups.

One-way ANOVA test was used to explore differences in traits [SLA, leaf nutrients (LCC, LNC, and LPC), C:N: and N:P stoichiometry, and PSI] among functional groups and shrublands. The chi-square (*X*^2^) test was used to assess the relationships between shrublands and functional groups and between drought avoidance mechanisms. The *t*-test was used to determine if the means of the coefficient of variation (CV) differed at all levels. In addition, means ± SD and CV of SLA, LNC, and LPC were calculated for drought-avoidance mechanisms.

The bivariate relationships of structural and nutrient leaf traits were first assessed with Pearson's correlation coefficient (*r*). When *r* was significant, regression analysis was applied to determine the relationship among SLA, leaf nutrients, and stoichiometry (*R*^2^, coefficient of linear regression expressed as LR). It is common for variables to be logarithmically transformed with the regression log (*y*)= log β + α log (x), which expresses a power law of the form *y* = β × α. The slope or scaling exponent α quantifies the rate of increase of *y* in relation to *x*, and is of special interest because it indicates the magnitude of the scaling between the variables. We excluded *Juniperus* from the bivariate relationships because gymnosperms were assessed differently from angiosperms (Diaz et al., 2016). ETs were excluded based on the low species number in the studied shrublands.

## Results

### Leaf Traits, Stoichiometry, and PSI Variation

**Figure 2:**
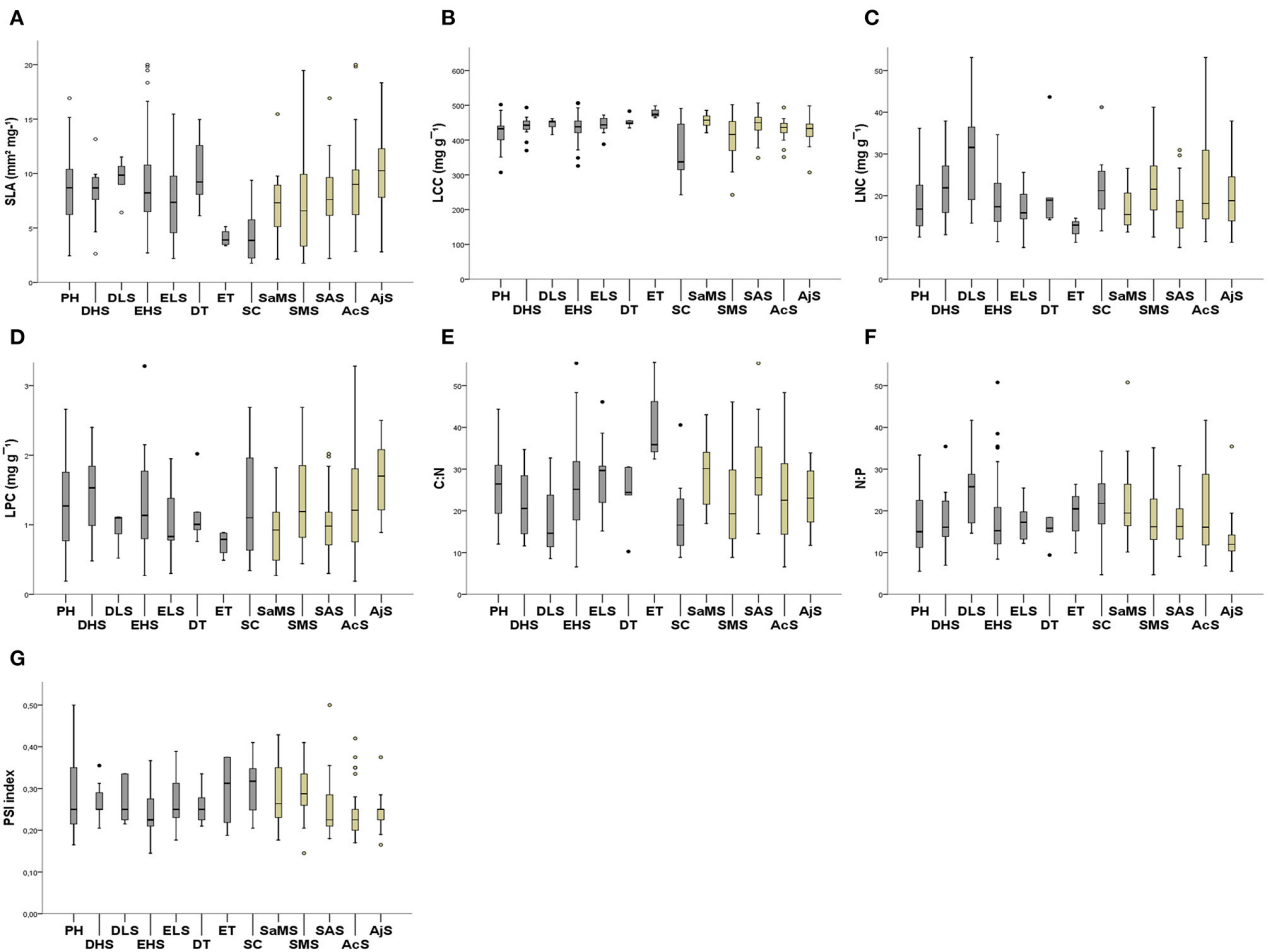
Mean trait values for the 126 perennial species grouped into functional groups and studied shrublands. **(A)** SLA; **(B)** LCC; **(C)** LNC; **(D)** LPC; **(E)** C:N; **(F)** N:P; and **(G)** PSI. Arid Mediterranean shrubland categories in brown: semiarid Mediterranean shrubland (SaMS), subdesert Mediterranean shrubland (SMS); subalpine shrubland (SAS); alpine cushion shrubland (AcS), alpine juniper prostrate shrubland (AjS). Functional groups categories in gray: evergreen trees (ETs); deciduous trees (DTs), evergreen large shrubs (ELSs); evergreen half shrubs (EHSs); deciduous large shrubs (DLSs); deciduous half shrubs (DHSs); succulents (SCs) and perennial herbs (PHs).

The mean and SD values for the studied traits at the species, shrubland (Table 1), and functional group levels (Table 2) are presented in Figure 2. All trait values are presented in Supplementary Material 1: Table S1.

**Table 1:** Leaf structural trait (SLA), leaf nutrient traits [leaf carbon content (LCC), leaf nitrogen content (LNC), and leaf phosphorous content (LPC)], stoichiometry (C:N and N:P), and phenophase sequence index (PSI) (a) for all species and within shrublands.

**Traits**	**Overall**		**SMS**		**SaMS**		**SAS**		**AcS**		**AjS**	
	**Mean ± SD**	**CV (%)**	**Mean**	**CV (%)**	**Mean**	**CV (%)**	**Mean**	**CV (%)**	**Mean**	**CV (%)**	**Mean**	**CV (%)**
SLA (mm^2^ mg^−^1)	8.3 ± 4	48	7.36 ± 6.6	90.1	7.2 ± 2.8	38.8	7.7 ± 3.2	41.5	9.1 ± 4.4	48.4	10.29 ± 4	38.4
LCC (mg g^−^1)	432.05 ± 43.0	10	401.36 ± 62.4	15.6	456 ± 18.5	4.1	446.2 ± 33.8	7.6	421.7 ± 28.5	6.8	426.1 ± 37.7	8.9
LNC (mg g^−^1)	19.8 ± 8.7	43.7	22 ± 8.3	37.9	16.6 ± 4.4	25.9	16.6 ± 5.7	34.1	23.6 ± 12.5	53.1	20.2 ± 8.0	39.6
LPP (mg g^−^1)	1.3 ± 0.8	58.6	1.5 ± 1.0	69.8	0.9 ± 0.4	46.7	1.0 ± 0.5	43.7	1.4 ± 0.9	63.2	1.7 ± 0.5	28.9
C:N (mass)	25.6 ± 10.4	40.7	21.3 ± 9.9	46.4	28.8 ± 7.7	26.9	30.1 ± 11.2	37.0	22.8 ± 10	43.8	24.3 ± 10.2	41.9
N:P (mass)	18.9 ± 1	58.2	17.8 ± 6.9	38.5	22.2 ± 9.4	42.2	17 ± 5.1	29.9	23.4 ± 18.7	79.4	13.2 ± 6.4	48.0
PSI index	0.5 ± 0.1	25.2	0.6 ± 0.1	21.1	0.6 ± 0.2	26.3	0.5 ± 0.13	26.0	0.5 ± 0.1	26.5	0.5 ± 0.1	16.7

*Mean value and SD (Mean ± SD), coefficient of variation (CV, defined as SD/Mean). SLA (mm^2^ mg^−1^), LCC, LNC, and LPC (mg·g^−1^), C:N, and N:P (on a dry mass basis). Arid Mediterranean Shrubland categories: Semiarid Mediterranean Shrubland (SaMS), Subdesert Mediterranean Shrubland (SMS); Subalpine Shrubland (SAS); Alpine Cushion Shrubland (AcS), Alpine Juniper prostrate Shrubland (AjS)*.

**Table 2:** Leaf structural trait (SLA), leaf nutrient traits (LCC, LNC, and LPC), stoichiometry (C:N and N:P), and PSI within the functional groups.

	**PH**		**DHS**		**DLS**		**DT**		**EHS**		**ELS**		**ET**		**SC**	
	**Mean ± SD**	**CV (%)**	**Mean ± SD**	**CV (%)**	**Mean ± SD**	**CV (%)**	**Mean ± SD**	**CV (%)**	**Mean ± SD**	**CV (%)**	**Mean ± SD**	**CV (%)**	**Mean ± SD**	**CV (%)**	**Mean ± SD**	**CV (%)**
SLA (mm^2^ mg^−1^)	8.5 ± 3.8	44.6	8.3 ± 2.5	30.3	9.5 ± 1.95	20.6	10.8 ± 2.9	26.55	9.2 ± 4.5	48.9	7.1 ± 3.5	49.7	4.47 ± 1.41	31.5	4.3 ± 2.6	59.7
LCC (mg g^−1^)	422.6 ± 40.8	9.7	437.9 ± 31.3	7.14	444.4 ± 18.4	4.2	446.9 ± 7.5	1.68	435.3 ± 36	8.3	446.3 ± 23	5.2	479.9 ± 14.5	3.0	366.2 ± 86	23.5
LNC (mg g^−1^)	18.1 ± 6.9	38.2	22.8 ± 8.8	38.49	30.7 ± 15.6	50.7	22.09 ± 12.26	55.50	22.8 ± 8.8	38.5	17.2 ± 6.8	39.3	14 ± 4.4	31.5	22.7 ± 9	40.1
LPC (mg g^−1^)	1.3 ± 0.6	44.62	1.5 ± 0.6	41.5	1.4 ± 1.2	86.1	1.14 ± 0.5	43.86	1.3 ± 0.6	48.0	1.2 ± 0.8	73.0	0.8 ± 0.3	30.5	1.6 ± 1.5	96.2
C:N (mass)	19.3 ± 16.6	86	22 ± 8.2	37.5	18.2 ± 9.9	54.4	23.6 ± 8.3	34.96	25.8 ± 9.9	38.4	29.9 ± 12.6	42	37.4 ± 13.5	36.2	21.2 ± 8.9	42
N:P (mass)	19.3 ± 16.6	86	17.4 ± 7.6	43.8	25.6 ± 10.7	41.9	23.1 ± 19.5	84.4	17.8 ± 8.6	48.2	17.9 ± 6.13	34.3	18.3 ± 16.6	90.67	21.1 ± 8.88	42.1
PSI index	0.6 ± 0.2	32.1	0.5 ± 0.8	154.7	0.5 ± 0.1	20.4	0.5 ± 1	192	0.5 ± 1	204.3	0.5 ± 0.1	22.6	0.6 ± 0.12	27.6	0.61 ± 0.1	21.3

*Mean value and SD (Mean ± SD), coefficient of variation (CV, defined as SD/Mean). SLA (m^2^ mg^−1^)), LCC, LNC, and LPC (mg·g^−1^), C:N, N:P (on dry mass basis). Functional groups categories: evergreen trees (ETs); deciduous trees (DTs), evergreen large shrubs (ELSs); evergreen half shrubs (EHSs); deciduous large shrubs (DLSs); deciduous half shrubs (DHSs); succulents (SCs), and perennial herbs (PHs)*.

**Figure 3:**
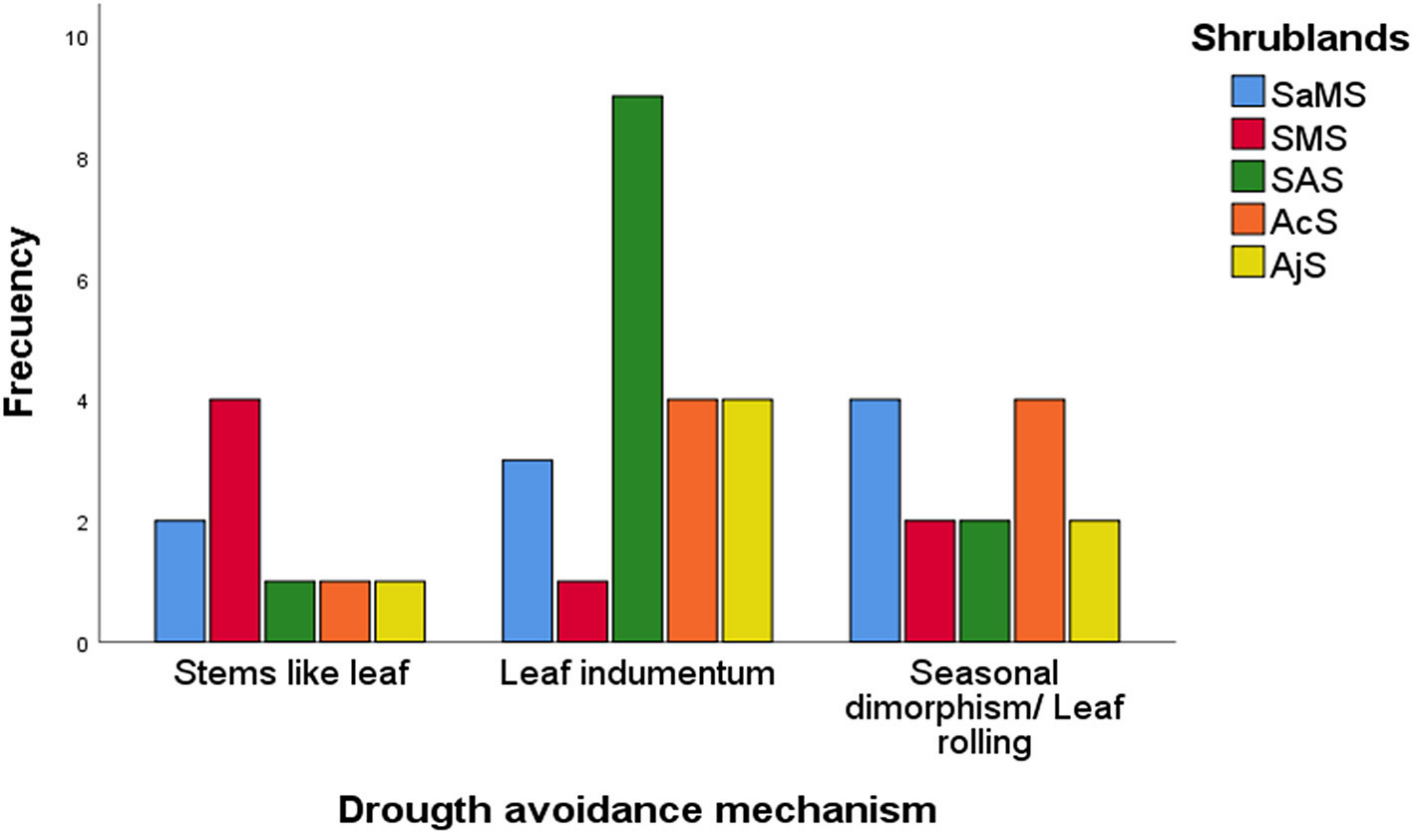
Frequency of drought-avoidance mechanisms according to Travlos and Chachalis (2008). SaMS, semiarid Mediterranean shrubland; SMS, subdesert Mediterranean shrubland; SAS, subalpine shrubland; AcS, alpine cushion shrubland; and AjS, alpine juniper prostrate shrubland.

A total of 44 species with different leaf drought avoidance mechanisms were observed within the evergreen large and half shrubs functional groups (see Supplementary Material 1: Table S1). The number of species with drought-avoidance mechanisms varied significantly between the studied shrublands (*X*^2^ = 22.098, *df* = 12, *p* = 0.036; Figure 3), but not between functional groups. Seasonal dimorphic species were present in all shrublands, and species with leaf indumentum were predominant in the Sierra Nevada mountain shrublands (SAS, AcS, and AjS). Within species with drought-avoidance mechanism, the highest SLA values were found in seasonal dimorphism species (11.2 ± 4.31 mm^2^·mg^−^1) followed by species with leaf indumentum (8.22 ± 5.0 mm^2^·mg^−^1) and stem-like leaves (8.4 ± 6.1 mm^2^·mg^−^1; refer to Supplementary Material 1: Table S2).

The CV of all traits across shrublands and functional groups (Tables 1, 2) showed significant differences (*t*-test, *p* < 0.05 and *p* < 0.01, respectively). For all species, the leaf traits presented a CV between 40 and 60%, except for LCC and the PSI (Table 1). Across studied shrublands, all leaf nutrient traits presented a CV <70% and across functional groups, they presented a CV <56%, except for C:N and N:P in PHs and N:P in ETs (Tables 1, 2). The range of variation of SLA between species within the studied shrublands was 38–48%, except for predesert shrublands (90%; Table 1). However, the range of variation of SLA between species within the studied functional groups was 20–60% (Table 2).

The range of variations (CV) of SLA, LNC and LPC for the studied species with drought-avoidance mechanisms is presented in Supplementary Material 1: Table S2.

Functional groups varied significantly among the studied shrublands (*X*^2^ = 76.153; *df* = 28; *p* < 0.05). The EHSs were predominant in alpine cushion and oro-Mediterranean heaths (25 and 31%, respectively), PHs were dominant only in oro-Mediterranean heaths (37.04%), whereas the SCs were dominant in predesert shrublands (87.5%). There were significant differences between the SLA and PSI indices among the studied shrublands (*F* = 3.64, *p* < 0.05; *F* = 3.00, *p* < 0.05, respectively) and functional groups (*F* = 2.97, *p* < 0.05; *F* = 1.671, *p* < 0.05) (Figures 2A,G; see Supplementary Material 3: Table S1B). SLA increased significantly with increasing altitude in Sierra Nevada shrublands (SAS, AcS, and AjS; Figure 2A; Table 1), and the PSI showed the same trend in the opposite manner (Figure 2G; Table 1). The value trend for SLA across the studied shrublands increased from predesert to semiarid and subalpine to alpine cushion to oro-Mediterranean heaths (Figure 2A); the same trends were observed for the PSI values (Figure 2G). The value trend for the SLA among functional groups increased from SCs to ETs and large shrubs, deciduous half shrub, PHs, EHSs to DTs (Figure 2A). With respect to the PSI, a lower degree of overlap between vegetative and reproductive growth occurred in SCs (Table S1 and Supplementary Material 2). We found significant differences between leaf nutrient traits and stoichiometry among all studied shrublands and among most functional groups (except for LPC and N:P; Figures 2D,F; see Supplementary Material 3: Tables S1, S2). LCC, LNC, and C:N varied significantly among the shrublands and functional groups (Figures 2B,C,E). Maximum and minimum leaf trait variation values among the studied shrublands and functional groups are indicated in Supplementary Material 1: Table S1.

### Relationships Between Leaf Traits, Stoichiometry, and PSI

Significant bivariate relationships were found between traits across all species, both at the studied shrublands and functional group levels. These relationships are not always consistent at different levels. In most cases, the relationships at the shrubland level were stronger than those at the functional group level. Pearson's correlation coefficients and *p*-values are indicated in Supplementary Material 4: Tables S1–S3.

**Figure 4:**
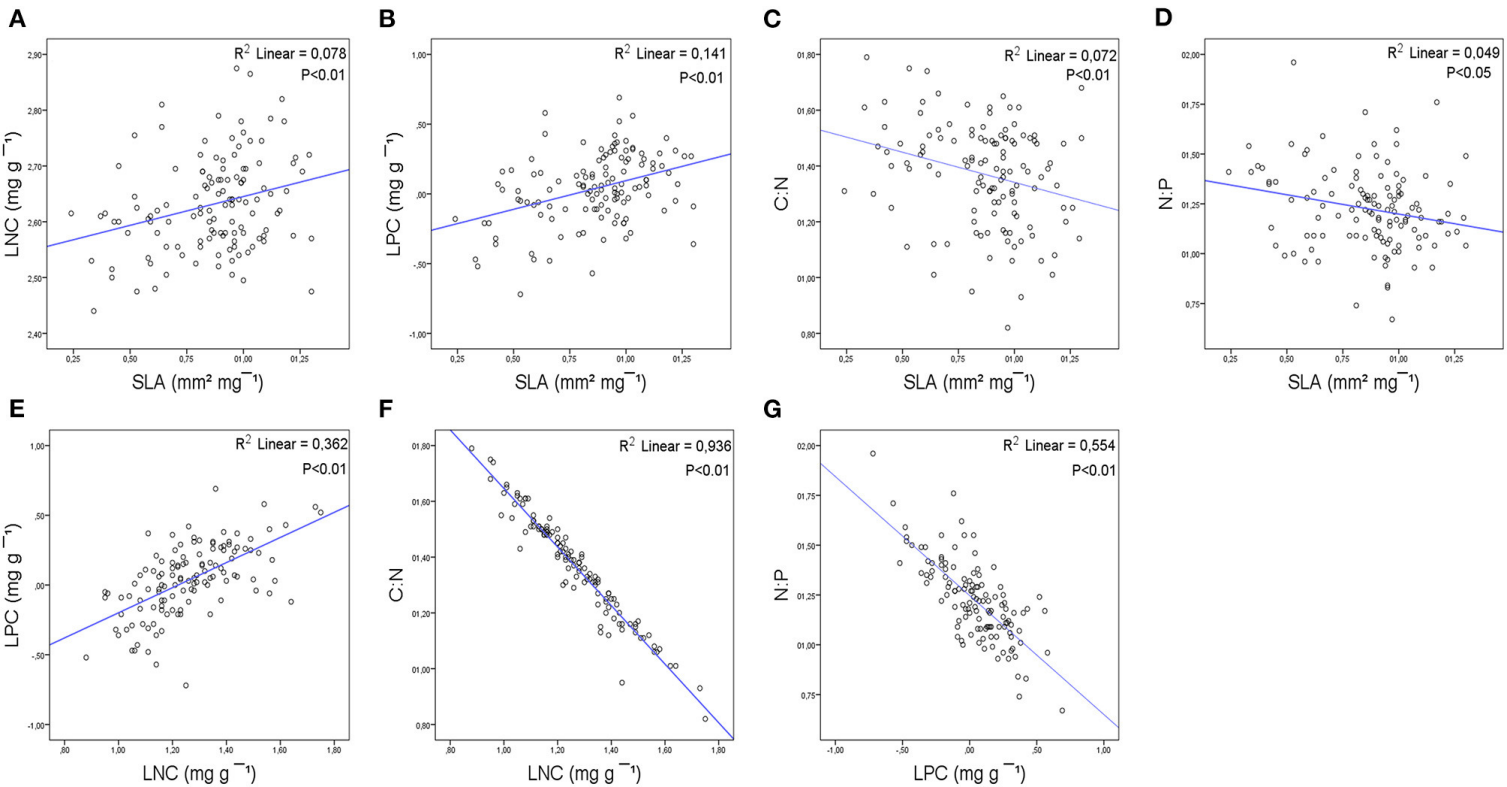
Bivariate relationships between leaf structural trait (SLA), leaf nutrient traits (LCC, LNC, and LPC), stoichiometry (C:N, N:P), and phenological index (PSI) at the species level based on linear regressions: **(A)** SLA-LNC; **(B)** SLA-LPC; **(C)** SLA-C:N; **(D)** SLA-N:P; **(E)** LPC-LNC; **(F)** LNC-C:N; **(G)** LPC-N:P. R^2^ coefficient of linear regression. *p*-values are indicated. Refer to Supplementary Material 4: Table S1 for a complete report of the results of the bivariate correlations.

For all species, SLA was positively correlated with LNC and LPC (Figures 4A,B) and negatively correlated with C:N and N:P (Figures 4C,D). LNC was positively correlated with LPC (Figure 4E) and negatively correlated with LCC (Supplementary Material 4: Table S1) and C:N (Figure 4F). LCC was negatively correlated with LPC and positively correlated with C:N (Supplementary Material 4: Table S1). LPC was negatively correlated with C:N (Supplementary Material 4: Table S1) and N:P (Figure 4G).

Some of these relationships were maintained completely or partially at the studied shrubland and functional group levels.

**Figure 5:**
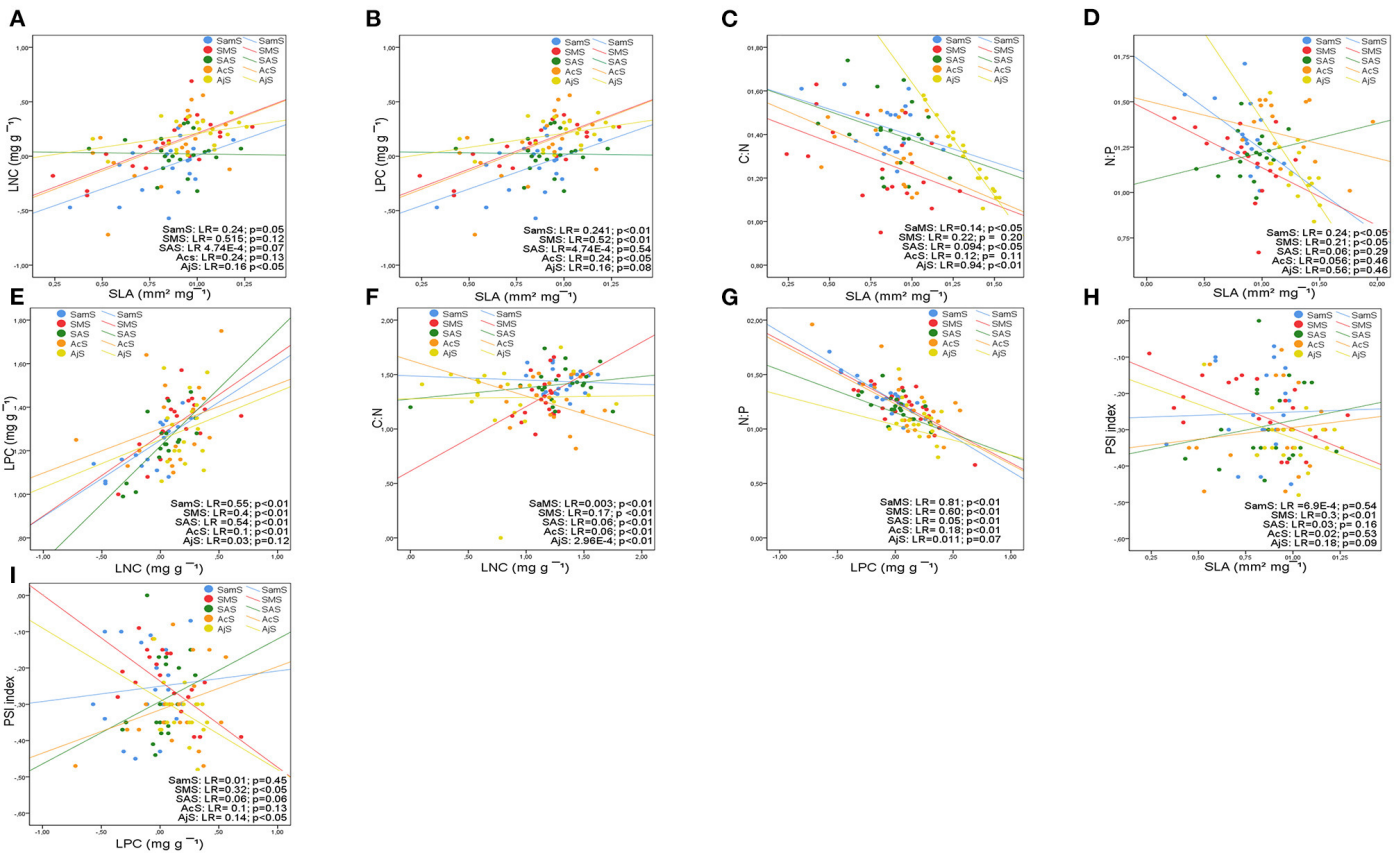
Bivariate relationships between leaf structural trait (SLA), leaf nutrient traits (LCC, LNC, and LPC), stoichiometry (C:N, N:P), and phenological index (PSI) at the studied shrubland level based on linear regressions: **(A)** SLA-LNC; **(B)** SLA-LPC; **(C)** SLA-C:N; **(D)** SLA-N:P; **(E)** LPC-LNC; **(F)** LNC-C:N; **(G)** LPC-N:P; **(H)** PSI-SLA; **(I)** PSI-LPC. Colors and symbols indicate the arid Mediterranean shrubland categories: semiarid Mediterranean shrubland (SaMS), subdesert Mediterranean shrubland (SMS); subalpine shrubland (SAS); alpine cushion shrubland (AcS), alpine juniper prostrate shrubland (AjS). LR= *R*^2^ coefficient of linear regression. *p*-values are indicated. See Supplementary Material 4: Table S2 for a complete report of the results of the bivariate correlations.

**Figure 6:**
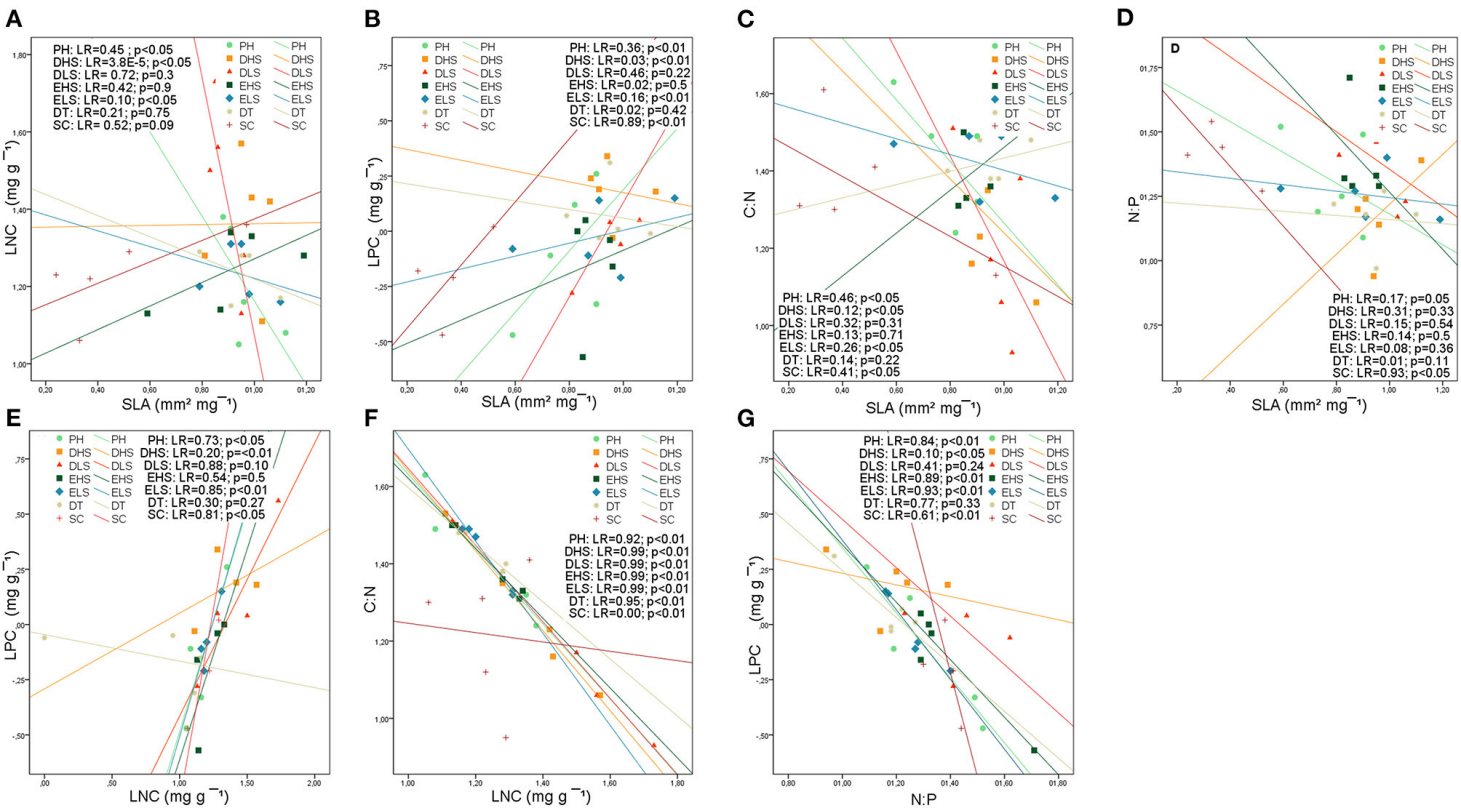
Bivariate relationships between leaf structural trait (SLA), leaf nutrient traits (LCC, LNC, LPC), stoichiometry (C:N, N:P), and phenological index (PSI) at the functional group level based on linear regression: **(A)** SLA-LNC; **(B)** SLA-LPC; **(C)** SLA-C:N; **(D)** SLA-N:P; **(E)** LPC-LNC; **(F)** LNC-C:N; **(G)** LPC-N:P. Colors and symbols indicate the arid Mediterranean shrubland categories. Deciduous trees (DTs); evergreen large shrubs (ELSs); evergreen half shrubs (EHSs); deciduous large shrubs (DLSs); deciduous half shrubs (DHSs); succulents (SCs), and perennial herbs (PHs). LR= *R*^2^ coefficient of linear regression. *p*-values are indicated. Refer to Supplementary Material 4: Table S3 for a complete report of the results of the bivariate correlations.

The most consistent relationship between SLA and leaf nutrient traits was with LPC at both levels, studied shrublands and functional groups (Figures 5B, 6B). The relationship between SLA and LPC was significantly positive in all studied shrublands, except for the subalpine shrublands, which showed stronger relationships (predesert and semiarid). At the functional group level, SCs, DHSs, ELSs, and perennial shrubs showed stronger correlations within the groups with the smallest leaves (SCs and DHSs) (Figure 6B). SLA was positively correlated with LNC only in the Sierra Nevada mountain shrublands (Figure 5A) and within functional groups in ELSs, deciduous half shrub, and PHs (Figure 6A). The negative relationships between SLA and C:N were maintained in all studied shrublands, except for the predesert and alpine cushion (Figure 5C) and at the functional group level, except for DTs, evergreen large and half shrubs (Figure 6C). However, the negative correlation between SLA and N:P was maintained only in the aridest shrublands (predesert and semiarid; Figure 5D) and SCs (Figure 6D). LNC and LPC positive relationships were maintained in all studied shrublands, except for the Sierra Nevada mountain shrublands (SAS, AcS, and AjS; Figure 5E); within the functional group, this positive relationship was only maintained for the evergreens and succulent functional groups (Figure 6E). LPC was consistently negatively correlated with N:P at the functional group level, except for DLSs (Figure 6G), and LNC was also negatively correlated with C:N at all functional group levels (Figure 6F). The most consistent relationships between PSI and leaf traits were negative for Sierra Nevada mountain shrublands with C:N (Supplementary Material 4: Table S2), N:P (Supplementary Material 4: Table S2), and LPC (Figure 5I), respectively. In predesert shrublands, PSI was positively correlated with SLA (Figure 5H) and with N:P (Supplementary Material 4: Table S2) and negatively correlated with LPC (Figure 5I).

## Discussion

### SLA Variation and Trends at the Shrubland and Functional Group Levels

In our dataset, the most variable traits were SLA and LPC, whereas the least variable was LCC, which is the most constant nutrient across environments indicating a high abundance of carbon content, in accordance with the results of De la Riva et al. (2018) and Fyllas et al. (2020).

It is known that SLA values vary with local environmental conditions and very particularly in relation to the soil fertility (Schulze et al., 2006) combining the increase in altitude due to linear augmentation of soil organic carbon, microbial biomass, and nitrogen soil concentration (He et al., 2016). This can explain the highest SLA values in the species from the Sierra Nevada mountain shrublands (SAS, AcS, and AjS). It should be noted that in these species the predominant drought-avoidance mechanism is the presence of leaf indumentum. An important role of the indumentum covering the leaf surface is the absorption of harmful UV-B radiation (Karabourniotis et al., 1992; Agati et al., 2012). The sensitivity to UV-B radiation is negatively correlated with the density of the indumentum, suggesting a UV-protective role especially in young leaves (Karabourniotis et al., 2020) which is of special interest to ensure the annual vegetative growth of plants. Thus, high SLA values help to modulate the phenological sequencing of plant growth. This modulation occurs through a clear reduction of the vegetative growth phase (Díaz et al., 2003) associated with the fast-growing species with high RGR (Reich et al., 1998) corroborating hypothesis number one of our study. SLA values decline substantially in predesert shrubland because SLA reduces transpiring leaf surfaces (Poorter et al., 2009) in the most drier environments (Xu et al., 2020) associated with leaf size reduction (Wright and Westoby, 2002), and, precisely, leaf reduction (leaf-like stem) is the predominant drought–avoidance mechanism for the predesert species (e.g., *Amarantaceae* family, *Launaea* genus and *Hammada* genus). There are two different viable solutions to water deficiency and drought (Turner, 1994).

### Leaf Nutrient Variation at the Shrubland and Functional Group Levels

The LNC and LPC values were relatively higher in Sierra Nevada mountain shrublands (SAS, AcS, and AjS) in accordance with the results of Körner et al. (1989), Körner (1999), and He et al. (2008). These mountain altitude shrublands are not nutrient-limited, their soil fertility stimulates plant overgrowth, concentrating vegetative and reproductive phases in two summer months. In accordance with the results of Han et al. (2011), all deciduous functional groups from these shrublands were mineral-rich leaves (high LNC, LPC, and LCC values) compared with the evergreen groups. Succulent was the functional group with more mineral-rich leaves in accordance with the results of Poorter et al. (2009), whereas the gymnosperm species was the lowest in accordance with the results of McGroddy et al. (2004) and Elser et al. (2010).

Although plants experiencing drought conditions tend to produce leaves with high LCC (Sardans and Peñuelas, 2012), our LCC was low compared with that found for other Mediterranean ecosystems (Hernández et al., 2011; Dominguez et al., 2012). This fact can be explained by the frequency of the different functional groups in this study; large trees and shrubs are less frequent than small shrubs according to Ma et al. (2016). LNC was higher than that reported by Hernández et al. (2011) and Dominguez et al. (2012) for woody plant species of the Mediterranean ecosystem, proximate to the mesic Mediterranean forest studied by Villar and Merino (2001), and lower than the xeric Mediterranean forest studied by the same authors, which is explained because our studied species, forming part of dry shrublands, are more adapted to drought which increases the LNC (Inclán et al., 2005; Sardans and Peñuelas, 2012). The values of LPC were higher than those reported by Dominguez et al. (2012) and Fyllas et al., 2020; see Supplementary Material 5: Table S1). This is consistent with the fact that a higher LPC improves the water use efficiency in dry studied shrublands, especially in the Sierra Nevada mountain shrublands.

The high LNC and LPC values detected in the species from Sierra Nevada mountain shrublands are associated with low PSI values, indicating that high LNC and LPC values can help in the overlapping of vegetative growth and reproduction phases, and consequently in the modulation of the phenological behavior. This corroborates the first hypothesis formulated in this study.

### Stoichiometry Variation at the Shrubland and Functional Group Levels

The N:P ratio in this study was based on the realistic range for the critical N:P ratio reported by Geider and La Roche (2002). The LNC and LPC scaling slopes vary considerably across functional groups and biomes (Tian et al., 2019). Extreme climatic conditions and the capacity of plants to store excess nutrients could also lead to the decoupling of LNC and LPC (He et al., 2008). When the LNC and LPC ratios found in our studied species are compared owing to limited growth caused by all nutrients (Güsewell, 2004; see Supplementary Material 5: Table S1), the LPC was found to be lower than the optimum. The N:P ratio had a slope of 0.363, indicating that P tended to accumulate at a slightly higher rate than N. This N:P ratio was found to be lower than the LES Global database (0.66; Wright et al., 2004; Reich et al., 2007), indicating that there is no high P limitation in the arid Mediterranean shrubland studied.

The stoichiometric values obtained for each shrubland reveal high variability in the N:P ratios, especially when Sierra Nevada mountain shrublands (SAS, AcS, and AjS) are compared with others. At elevations, plants retain nutrients better during the winter (Güsewell, 2004), contributing to the lower N:P values. Koerselman and Meuleman (1996) suggested that the N:P ratio indicates P or N limitation. Therefore, an N:P ratio less than 14 (100:7) indicates N limitation whereas an N:P ratio greater than 16 (100:6) indicates P limitation. Güsewell and Koerselman (2002) found that N limitation could not be clearly shown for N:P ratios lower than 14 (100:7). These results suggest that only subalpine and alpine cushion shrublands from Sierra Nevada mountain present P limitations. Differences in N:P among habitats are mainly associated with P variations as N is often the nutrient-limiting growth factor (Vitousek and Howarth, 1991; Soudzilovskaia et al., 2005).

In this study, all deciduous species and PHs had high C:N values, which reflect conservative mechanisms associated with increasing drought (Sardans and Peñuelas, 2012) and with an overlap of vegetative and reproductive phases to a restriction in resource remobilization throughout the year (Sardans et al., 2008).

### Leaf Traits, PSI, and Species With Drought-Avoidance Mechanisms

The high variation in SLA and LPC could be explained according to the coexistence of plant mechanisms favoring drought-avoidance vs. drought-resistance strategies (e.g., PHs, deciduous, and semideciduous species vs. SCs and evergreens). The specialized strategies of drought-avoidance vs. resistance that would have been competitively excluded in less severe environments (Freschet et al., 2011) were found to co-occur in our study area. This is the case for SCs, which dramatically increase the variation in SLA and LPC [Poorter et al. (2009)], and is the case for several species with drought-avoidance mechanisms (Travlos and Chachalis, 2008), which clearly contribute to the increase in mean SLA. For example, species with seasonal dimorphism and/or leaf indumentum have higher SLA than those without these features that belong to the same functional group. ETs, which mainly include sclerophyllous species, showed the lowest SLA, LNC, and LPC, in accordance with the results of Fyllas et al. (2020).

Semideciduous species (70% of the EHSs) partly shed their leaves during summer to reduce water loss (Tavşanoglu and Pausas, 2018). This species shows some similar patterns to deciduous species, such as the highest SLA and LNC, and lowest PSI (0.45) needing nutrient requirements throughout the seasons, corroborating hypothesis number two of this study. These results support the common leaf nutrient use strategies associated with leaf habit and water stress avoidance adaptations (Sardans and Peñuelas, 2013), showing that in the Mediterranean shrublands, where sclerophyllous species are less frequent (Parsons, 1976), other morphological and phenological traits related to water stress avoidance could increase when drought increases without significant losses in production and survival. This is the case, as this study shows, of seasonally dimorphic (semideciduous) species, one of the dominant growth forms in Mediterranean ecosystems (Puglielli, 2019).

### Trends in the Mediation of SLA and Leaf Nutrients in the Vegetative and Reproductive Phenological Sequencing in Predesert and Oro-Mediterranean Shrublands

The lowest PSI and the highest SLA, LPC, and LNC values were found in the Sierra Nevada mountains shrublands, whereas the highest PSI and lowest SLA were found in predesert shrublands, demonstrating the importance of the ranges of variation of the SLA and leaf nutrients in the modulation of the vegetative and reproductive phenological sequencing in arid Mediterranean shrublands in accordance with the first hypothesis formulated in this study.

Species from the Sierra Nevada mountain showed a high overlap of phenophases. These species concentrate on both vegetative and reproductive growth during the summer (refer to Supplementary Material 2), adopting a fast-growing strategy (Freschet et al., 2011). Their vegetative and reproductive demands must be supplied almost simultaneously in a mean of 2.85 months, establishing a competition for resources (Mooney, 1983). Growing and flowering occur when resources are abundant and the more productive habitats remain with low phenological activity during the rest of the year, and high SLA aids in this fast-growing strategy. On the contrary, the correlation between SLA and PSI was significant for sequential species found in predesert shrublands with low SLA and high PSI, which minimize intraplant competition growth in habitats where resources are scarce. These species maintain vegetative growth continuously for a mean of 5.9 months and synchronize vegetative growth and reproduction in the winter. The predesert and semiarid shrublands with low SLA overlap their vegetative and reproductive phases in winter or early spring, extending vegetative growth continuously for approximately 6 months. These results corroborate our hypothesis number one.

In the Sierra Nevada mountain shrublands, the association between the most favorable temperature during the short growing summer and the relatively large amounts of nutrients released in spring increases the productivity of these systems, which seems to drive high mean LNC and SLA as per Körner et al. (1989). This productivity also increases due to the ecological effect of plant facilitation studied in these ecosystems and based on plant aggregated spatial patterns (Alados et al., 2017). However, the high diversity of growth forms with special drought adaptations also contributes to the increase in SLA mean, such as PHs (70% of the total; e.g., large basal rosette leaves like *Jasione*) and the EHSs (72% of the total; e.g., stem-like leaves; *Hormathophylla* and a seasonally dimorphic *Teucrium*) in accordance with the formulated hypothesis number two. In these species, the high SLA allows high phenological synchronization (overlapping) in vegetative growth and flowering (mean of 2.85 months; PSI: refer to Supplementary Material 2) helping them to modulate their phenology in response to environmental conditions.

### Leaf Traits, Stoichiometry, and PSI Relationships Within Species, Shrublands, and Functional Groups

Trait covariations are useful for identifying functional trade-offs and plant strategies (Fyllas et al., 2020). Consistently positive correlations were found between SLA and LNC and LPC in the 126 perennial species studied in accordance with the results of Wright et al. (2004, 2005) and Liu et al. (2010). These results align with the leaf economic spectrum and corroborate hypothesis number three of this study. Species with high SLA tend to have higher LNC and higher photosynthetic N use efficiency (LPC) (Reich et al., 1998; Wright et al., 2005).

Specific leaf area with LNC and LPC was found to be highly correlated among all our datasets, in accordance with the results of Reich et al. (1997). The relationships between leaf structure and nutrient traits were stronger at the studied shrubland level than at the functional group level, in accordance with the results of Dominguez et al. (2012). The general trade-off disappeared partially when species growing under similar ecological filters were considered (Fyllas et al., 2009; De la Riva et al., 2018); this occurs within species from the same shrubland due to the broad floristic range. However, the SLA and LPC correlations were consistently maintained at the shrubland and functional group levels only within PHs, DHSs, and SCs. These species groups show higher LNC and SLA than evergreen species (Cornelissen et al., 1997) and increase carbon in nutrient cycling, accelerating ecosystem productivity. The SLA, LNC, and LPC correlations were only maintained in the oro-Mediterranean heath. The range in SLA was larger than the LNC range among the functional groups, indicating that SLA can be considered the main leaf trait associated with leaf habit in perennial plants (Poorter and van der Werf, 1998). Therefore, SLA can be considered a key variable for explaining the differences in leaf phenology, including semideciduous species, and in modulation of vegetative and reproductive synchronization in Mediterranean arid shrublands in accordance with the hypotheses number one and two formulated in this study.

Specific leaf area was negatively correlated with C:N among all datasets and was maintained consistently at the studied shrubland level and partially at the functional group level. Based on this correlation, plant investment in structure (LCC) and functioning (LNC) disappears partially when species functional groups are considered (only maintained in SCs and ELSs). Species that possess leaves with higher C:N ratios are usually slow-growing (Villar et al., 2006) and show a prolonged vegetative growth over the mean found in the overall dataset. SLA and net photosynthetic capacity may be more closely linked to P (Liu et al., 2010). Indeed, we found a significant positive correlation between SLA and LPC in the overall dataset and in all shrublands (only negative for the oro-Mediterranean heath), where low temperatures and highest precipitation increased the LPC (Sardans et al., 2011).

Leaf nitrogen content and LPC typically correlate with each other (Güsewell, 2004) depending on the availability of N and P in the soil (Güsewell and Koerselman, 2002). Consequently, a positive correlation was found between LNC and LPC in the overall dataset; this was maintained within all studied shrublands, corroborating our hypothesis number three, except for the Sierra Nevada high mountains. The significant positive correlation between LNC and LPC at the studied shrubland level suggests that these traits have similar patterns across the arid Mediterranean shrublands that determine species assembly community within sites.

## Conclusion

In summary, the findings of this study confirm that arid Mediterranean shrublands have the main global variation in SLA among habitats and plant functional groups, including the existence of the leaf economics spectrum in a broad pool of Mediterranean perennial species. We revealed the importance of SLA and leaf nutrients in the modulation of plant vegetative and reproductive phenological sequencing in extreme climatic habitats. High SLA values are often associated with the overlapping in growth and reproductive phases and a seasonal reduction of vegetative growth, whereas low SLA values are associated with vegetative and reproductive sequencing and a seasonal extension of the vegetative growth phase. The N:P ratio found to indicate that P tended to accumulate at a slightly higher rate than N and that, only the alpine mountain shrublands, could present P limitations. Deciduous and PHs studied had high C:N values as conservative mechanisms associated with increasing drought. Further, seasonal dimorphic species, which are predominant in Mediterranean ecosystems affected by drought, were identified as the main driver of variability in SLA and LPC. This finding corroborates the notion that SLA is a proxy of the growth and abundance of the plants in Mediterranean arid shrublands and may help to predict ecosystem functions under future climate scenarios. In addition, we provide relevant database information on leaf traits and stoichiometry in arid Mediterranean shrublands.

## Data Availability Statement

The original contributions presented in the study are included in the article/Supplementary Material, further inquiries can be directed to the corresponding authors.

## Author Contributions

Both authors listed have made a substantial, direct and intellectual contribution to the work, and approved it for publication.

## Conflict of Interest

The authors declare that the research was conducted in the absence of any commercial or financial relationships that could be construed as a potential conflict of interest.

## Publisher's Note

All claims expressed in this article are solely those of the authors and do not necessarily represent those of their affiliated organizations, or those of the publisher, the editors and the reviewers. Any product that may be evaluated in this article, or claim that may be made by its manufacturer, is not guaranteed or endorsed by the publisher.
