# Supplementary material for: Variations in Leaf Traits Modulate Plant Vegetative and Reproductive Phenological Sequencing Across Arid Mediterranean Shrublands
Source: Front Plant Sci. 2021 Aug 23;12:708367. doi: 10.3389/fpls.2021.708367 (PMC8420881; doi:10.3389/fpls.2021.708367)
Supplement: Supplementary file 1 [file Data_Sheet_1.PDF]

Supplementary Material 1

Table S1. Studied traits for the 126 perennial species. Families follows Blanca et al. (2011). Arid Mediterranean Shrubland formations categories: Semiarid Mediterranean Shrubland (SaMS). Subdesert Mediterranean Shrubland (SMS); Subalpine Shrubland (SAS); Alpine Cushion Schrubland (AcS). Alpine Juniper prostrate Shrubland (AjS). Functional groups categories: Evergreen Trees (ET); Deciduous Trees (DT). Evergreen Large Shrubs (ELS); Evergreen Half Shrubs (EHS); Deciduous Large Shrubs (DLS); Deciduous Half Shrubs (DHS); Succulents (SC) and Perennial Herbs (PH). Drought avoidance mechanism following Travlos & Chachalis (2008). Specific leaf area (SLA). LCC. LNC and LPC (leaf carbon. leaf nitrogen and leaf phosphorous content respectively). leaf C:N:P stoichiometry. and Phenological index (PSI). Endemic species (*).

ID	Taxa	Family	Mediterranean formation	Functional groups	Drought avoidance	SLA (mm^2 mg^{-1})	PSI index	C : N (mg g^{-1})	N: P (mgg $^{-1}$)	C (mg g $^{-1}$)	N (mg g $^{-1}$)	P (mg g $^{-1}$)
1	*Artemisia herba-alba* Asso	Asteraceae	SaMS	DHS	-	8.18	0.5	17	17.2852	458.6	26.55	1.54
2	*Asparagus acutifolius* L.	Asparagaceae	SaMS	PH	-	8	0.8571	20.8256	12.3655	469.2	22.53	1.82
3	*Asparagus albus* L.	Asparagaceae	SaMS	PH	-	8	0.8	30.5907	31.2446	445.4	14.56	0.47
4	*Asparagus horridus* L.	Asparagaceae	SaMS	PH	-	3.88	0.8	43.023	33.3728	485.3	11.28	0.34
5	*Ballota hirsuta* Benth.	Lamiaceae	SaMS	EHS	Seasonal dimorphism/leaf rolling	9.06	0.7333	31.8087	19.438	429.1	13.49	0.69
6	*Chamaerops humilis* L.	Arecaceae	SaMS	ELS	-	3.87	0.7778	29.6669	19.1687	472	15.91	0.83
7	*Cistus albidus* L.	Cistaceae	SaMS	ELS	Leaf indument	7.36	0.5	30.6432	18.634	443.1	14.46	0.78
8	*Cistus salviifolius* L.	Cistaceae	SaMS	ELS	None	15.46	0.6667	21.5019	14.5866	443.8	20.64	1.42
9	*Coris monspeliensis* L.	Cistaceae	SaMS	EHS	None	7.04	0.5	31.912	50.7692	442.3	13.86	0.27
10	*Daphne gnidium* L.	Thymelaceae	SaMS	ELS	None	9.77	0.3529	30.7176	24.835	462.3	15.05	0.61
11	*Genista spartioides* Spach	Fabaceae	SaMS	SC	Stems like leaf	2.13	0.4545	40.5791	34.3323	469.5	11.57	0.34
12	*Lavatera maritima* Gouan	Malvaceae	SaMS	ELS	Leaf indument	8.22	0.4615	20.6932	14.7285	420.9	20.34	1.38
13	*Olea europaea* L.	Oleaaceae	SaMS	ET	-	6.12	0.5556	24.8329	16.4413	483	19.45	1.18
14	*Phlomis lychnitis* L.	Lamiaceae	SaMS	EHS	Leaf indument	8.93	0.5556	22.8482	21.1356	438	19.17	0.91
15	*Phlomis purpurea* L.	Lamiaceae	SaMS	ELS	Seasonal dimorphism/leaf rolling	4.56	0.5	23.578	17.2676	433.6	18.39	1.07
16	*Quercus coccifera* L.	Fagaceae	SaMS	ET	-	5.12	0.375	35.861	26.3747	464.4	12.95	0.49
17	*Rhamnus lycioides* L.	Rhamnaceae	SaMS	ELS	None	6.91	0.5	22.0203	19.7612	455.6	20.69	1.05
18	*Ruta chalepensis* L.	Rutaceae	SaMS	EHS	None	6.79	0.375	20.4582	21.0947	437.6	21.39	1.01
19	*Teucrium charidemi* Sandwith	Lamiaceae	SaMS	EHS	Seasonal dimorphism/leaf rolling	7.25	0.7	21.572	19.517	470.7	21.82	1.12
20	*Teucrium lusitanicum* Schreb.	Lamiaceae	SaMS	EHS	Seasonal dimorphism/leaf rolling	9.49	0.46	40.5705	10.1361	483.6	11.92	1.18
21	*Thymus hyemalis* Lange	Lamiaceae	SaMS	DHS		9.06	0.625	34.0015	13.8511	442.7	13.02	0.94
22	**Ulex canescens* Lange	Fabaceae	SaMS	EHS	Stems like leaf	3.76	0.4444	39.0793	31.7886	458.4	11.73	0.37
				MeanSaMS	-	7.23	0.58	28.06	23.11	452.93	17.27	0.90
				Variance	-	7.89	0.02	61.85	115.12	501.09	19.55	0.19
				Std. Desviation	-	2.81	0.15	7.86	10.73	22.39	4.42	0.43
				Minimum	-	2.13	0.35	17.00	10.14	390.30	11.28	0.27
				Maximum	-	15.46	0.86	43.02	50.77	485.30	26.55	1.82

				Range	-	13.33	0.50	26.02	40.63	95.00	15.27	1.55
				n	22	22	22	22	22	22	22	22
23	*Helianthemum almeriense* Pau	Cistaceae	SMS	DHS	-	8.68	0.41	22.54	8.75	432.50	19.19	2.19
24	*Anthyllis cytisoides* L.	Fabaceae	SMS	DHS	-	13.16	0.48	11.5984	24.4428	433.2	37.35	1.53
25	*Lycium intricatum* Boiss.	Solanaceae	SMS	SC	None	9.38	0.41	13.3463	4.6978	308.3	23.1	4.92
26	**Thymus baeticus* Boiss. ex Lacaita	Lamiaceae	SMS	EHS	Seasonal dimorphism/leaf rolling	9.09	0.6	31.3407	14.4371	453.5	14.47	1
27	*Teucrium lusitanicum* Schreb.	Lamiaceae	SMS	EHS	Seasonal dimorphism/leaf rolling	13.21	0.29	22.8742	12.1956	460	20.11	1.65
28	*Thymelaea hirsuta* (L.) Endl.	Thymelaceae	SMS	ELS	None	4.53	0.67	46.1057	12.4112	471.2	10.22	0.82
29	*Launaea lanifera* Pau	Asteraceae	SMS	EHS	Stems like leaf	19.47	0.55	13.7685	14.9821	380.7	27.65	1.85
30	**Vella bourgaeana* (Coss.) Warwick & Al-Shehbaz	Brassicaceae	SMS	EHS	None	7.14	0.45	14.1645	16.6759	325.5	22.98	1.38
31	*Launaea arborescens* (Batt.) Murb.	Asteraceae	SMS	ELS	Stems like leaf	11.79	0.41	15.1681	13.1391	388	25.58	1.95
32	**Limonium majus*(Boiss.) Erben	Plumbaginaceae	SMS	PH	None	5.36	0.7	30.9182	15.532	370.4	11.98	0.77
33	*Lavandula multifida*L.	Lamiaceae	SMS	DHS	-	7.62	0.52	14.4952	15.7314	393.4	27.14	1.73
34	*Salsola webbii* Moq.	Chenopodiaceae	SMS	SC	-	2.33	0.58	19.8252	27.2713	328.9	16.59	0.61
35	*Cistus clusii* Dunal	Cistaceae	SMS	SC	-	3.3	0.7	25.4141	18.4588	491	19.32	1.05
36	*Frankenia corymbosa*Desf.	Frankeniaceae	SMS	DHS	-	2.63	0.61	34.6904	22.3422	369.8	10.66	0.48
37	*Plantago lanceolata* L.	Plantaginaceae	SMS	PH	-	6.66	0.54	17.2739	17.9772	410.6	23.77	1.32
38	*Carlina hispanica* Lam.	Asteraceae	SMS	PH	-	10.18	0.64	21.1879	19.7539	392.4	18.52	0.94
39	*Limonium insigne* (Coss.) Kuntze	Plumbaginaceae	SMS	PH	-	2.64	0.53	42.3861	22.8414	428.1	10.1	0.44
40	*Anabasis articulata* (Forssk.) Moq.	Chenopodiaceae	SMS	SC	-	1.76	0.82	20.3055	25.7328	345.6	17.02	0.66
41	*Salsola papillosa* (Coss.) Willk.	Chenopodiaceae	SMS	SC	-	6.49	0.69	8.8504	23.7777	242.5	27.4	1.15
42	*Salsola oppositifolia* Desf.	Chenopodiaceae	SMS	SC	-	4.99	0.69	13.2442	19.7712	322.1	24.32	1.23
43	*Artemisia herba-alba*Asso	Asteraceae	SMS	DHS	-	9.93	0.58	18.801	10.34	465.7	24.77	2.4
44	*Haloxylon tamariscifolium* (L.) Pau	Chenopodiaceae	SMS	EHS	Stems like leaf	3.32	0.57	12.9769	35.1001	421.1	32.45	0.92
45	*Salsola genistoides*Juss. ex Poir.	Chenopodiaceae	SMS	SC	-	4.4	0.54	10.2354	15.3058	421.8	41.21	2.69
46	*Retama sphaerocarpa* (L.) Boiss.	Fabaceae	SMS	EHS	Stems like leaf	4.36	0.62	13.132	9.1784	454.5	34.61	3.77
47	*Asparagus horridus* L.	Asparagaceae	SMS	PH	-	2.44	0.79	29.7864	26.9851	501.9	16.85	0.62
48	*Phagnalon saxatile* (L.) Cass.	Asteraceae	SMS	EHS	Leaf indument	16.53	0.55	30.0569	15.678	422.9	14.07	0.9
				MeanSMS	-	7.36	0.57	21.33	17.83	401.37	21.98	1.50
				Variance	-	7.89	0.02	98.20	47.07	3887.76	69.27	1.08
				Std. Desviation	-	2.81	0.12	9.91	6.86	62.35	8.32	1.04
				Minimum	-	1.76	0.29	8.85	4.70	242.50	10.10	0.44
				Maximum	-	19.47	0.82	46.11	35.10	501.90	41.21	4.92
				Range	-	17.71	0.53	37.26	30.40	259.40	31.11	4.48
				n	26	26	26	26	26	26	26	26
49	*Anthyllis vulneraria* subsp. *arundana (Boiss. & Reut.) Vasc.*	Fabaceae	SAS	PH	-	6.61	0.67	15.84	30.8	377.2	23.82	0.77
50	*Artemisia granatensis* Boiss.	Asteraceae	SAS	EHS	Leaf indument	8.51	0.6	18.77	11.43	424.1	22.59	1.98
51	*Berberis hispanica* Boiss. & Reut	Berberidaceae	SAS	DLS	-	11.52	0.67	23.75	17.11	453.1	19.08	1.11

52	*Bupleurum fruticenscens* L.	Apiaceae	SAS	EHS	None	6.5	0.64	26.86	14.94	453.9	16.9	1.13
53	*Cotoneaster granatensis* Boiss.	Rosaceae	SAS	DT	-	12.58	0.67	30.42	14.97	446.3	14.67	0.98
54	*Crataegus monogyna* Jacq.	Rosaceae	SAS	DT	-	8.08	0.45	30.54	15.23	434.9	14.24	0.93
55	*Daphne oleoides* Schreb.	Thymelaceae	SAS	ELS	None	6.43	0.625	26.12	12.22	461.8	17.68	1.45
56	*Digitalis purpurea* L. subsp. *purpurea*	Veronicaceae	SAS	PH	-	8.9	0.4	44.35	16.64	459	10.35	0.62
57	*Erinacea anthyllis* Link subsp. *anthyllis*	Fabaceae	SAS	DHS	-	9.63	0.71	14.53	16.08	430.9	29.66	1.84
58	*Euphorbia nicaeensis* All. subsp. *nicaeensis*	Euphorbiaceae	SAS	PH	-	16.92	0.44	15.82	22.5	420.9	26.61	1.18
59	*Helianthemum apenninum* subsp. *estevei (Peinado & Mart. Parras) G. López*	Cistaceae	SAS	DHS	-	7.45	0.5	26.19	16.26	423.3	16.16	0.99
60	*Helichrysum stoechas* (L.) Moench	Asteraceae	SAS	EHS	Leaf indument	4.12	0.39	55.38	10.54	506.7	9.15	0.87
61	*Helleborus foetidus* L.	Ranunculaceae	SAS	EHS	None	4.13	0.5	25.25	12.24	436.9	17.3	1.41
62	*Jurinea humilis*(Desf.) DC.	Asteraceae	SAS	EHS	Leaf indument	6.76	0.45	14.53		449.5	30.94	
63	*Lavandula lanata* Boiss.	Lamiaceae	SAS	EHS	Leaf indument	6.15	0.45	41.71	21.87	465.5	11.16	0.51
64	*Linum narbonense* L.	Linaceae	SAS	EHS	None	7.07	0.36	23.01	19.1	400.8	17.42	0.91
65	*Lonicera arborea* Boiss.	Caprifoliaceae	SAS	EHS	None	11.07	0.45	25.21	15.49	428.6	17	1.1
66	*Onosma tricerosperma* Lag.	Boraginaceae	SAS	EHS	Leaf indument	9.93	0.43	35.29	20.55	348.7	9.88	0.48
67	*Phlomis crinita* Cav.	Lamiaceae	SAS	EHS	Leaf indument	2.7	0.42	28.38	13.57	452.3	15.94	1.17
68	*Prunus mahaleb* L.	Rosaceae	SAS	DT	-	9.46	0.42	23.8	18.42	449.9	18.9	1.03
69	*Prunus ramburii* Boiss.	Rosaceae	SAS	DT	-	8.98	0.5	23.99	9.41	455.3	18.98	2.02
70	*Quercus ilex* subsp. *ballota* (Desf.) Samp	Fagaceae	SAS	ET	-	4.19	0.5	32.42	20.49	473.7	14.61	0.71
71	*Rhamnus saxatilis* Jacq.	Rhamnaceae	SAS	ELS	Leaf indument	10.09	0.57	30.06	22.13	436.8	14.53	0.66
72	*Rosa canina* L.	Rosaceae	SAS	ELS	None	7.75	0.5	38.63	13.22	423	10.95	0.83
73	*Salvia lavandulifolia* Vahl	Lamiaceae	SAS	EHS	Leaf indument	7.22	0.42	34.55	18.81	505.5	14.63	0.78
74	*Santolina rosmarinifolia* L.	Asteraceae	SAS	EHS	Leaf indument	3.88	0.5	28.06	12.21	469.7	16.74	1.37
75	*Santolina chamaecyparissus* L.	Asteraceae	SAS	EHS	Leaf indument	3.8	0.41	27.84	9.05	448.5	16.11	1.78
76	*Teucrium similatum* T.Navarro & Rosua	Lamiaceae	SAS	EHS	Seasonal dimorphism/leaf rolling	10.52	0.44	40.29	23.36	492.7	12.23	0.52
77	*Thymus mastichina* (L.) L.	Lamiaceae	SAS	EHS	Seasonal dimorphism/leaf rolling	7.77	0.36	40.89	17.81	486.2	11.89	0.67
78	*Ulex parviflorus* Pourr.	Fabaceae	SAS	ELS	Stems like leaf	2.2	0.36	61.77	25.47	468.8	7.59	0.3
				MeanSAS	-	7.70	0.50	30.22	17.19	444.89	16.41	1.01
				Variance	-	10.18	0.02	118.16	25.24	1267.16	30.54	0.20
				Std. Desviation	-	3.19	0.14	10.87	5.02	35.60	5.53	0.45
				Minimum	-	2.20	0.36	14.53	9.05	348.70	7.59	0.30
				Maximum	-	16.92	1.00	61.77	30.80	506.70	30.94	2.02
				Range	-	14.72	0.90	47.24	21.75	158.00	23.35	1.72
				n	30	30	30	30	29	30	30	29
79	*Acinos alpinus* subsp. *meridionalis* (Nyman) P. W. Ball	Lamiaceae	AcS	PH	-	12.23	0.4	32.7	10.62	441.4	13.5	1.27
80	*Alyssum serpyllifolium* Desf.subsp. *sepyllifolium*	Brassicaceae	AcS	PH	-	8.97	0.7	19.41	6.8	351	18.08	2.66

81	*Anthyllis vulneraria* subsp. *arundana* (Boiss. & Reut.) Vasc.	Fabaceae	AcS	PH	-	8.69	0.84	32.06	9.65	400.8	12.5	1.3
82	*Artemisia granatensis* Boiss.	Asteraceae	AcS	EHS	Leaf indument	8.08	0.7	18.68	12.87	460.7	24.66	1.92
83	*Astracantha granatensis* (Lam.) Podlech	Fabaceae	AcS	DHS	-	4.64	0.43	31.44	23.79	493.6	15.7	0.66
84	*Berberis hispanica* Boiss. & Reut	Berberidaceae	AcS	DLS	-	10.66	0.67	8.55	14.64	454	53.12	3.63
85	*Cerastium alpinum* var. *nevadense* Pau	Caryophyllaceae	AcS	PH	-	13.98	0.56	24.59	8.56	415.1	16.88	1.97
86	*Chaenorhinum glareosum* (Boiss.) Willk.	Veronicaceae	AcS	PH	-	6.03	0.56	26.08	12.31	436.6	16.74	1.36
87	*Dianthus pungens* subsp. *brachyanthus* (Boiss.) B.Fern.Casas. G.López & M.Laínz	Caryophyllaceae	AcS	PH	-	3.09	0.45	30.39	9.83	440.3	14.49	1.47
88	*Draba hispanica* Boiss.	Brassicaceae	AcS	PH	-	9.2	0.34	14.4	11.83	399.6	27.75	2.35
89	*Erinacea anthyllis* Link subsp. *anthyllis*	Fabaceae	AcS	DLS	-	6.42	0.43	32.67	25.78	438.4	13.42	0.52
90	*Euphorbia nicaensis*All.	Euphorbiaceae	AcS	PH	-	10	0.45	12.88	19.52	424.4	32.94	1.69
91	*Genista versicolor* Boiss.	Fabaceae	AcS	DLS	-	9	0.5	14.63	28.76	461.8	31.57	1.1
92	*Helianthemum apenninum* subsp. *stoechadifolium* (Brot.) Samp.	Cistaceae	AcS	DHS	-	9.84	0.5	20.57	14.95	450.3	21.89	1.46
93	*Hormathophylla spinosa*(L.) P. Küpfer	Brassicaceae	AcS	EHS	Stems like leaf	9.23	0.45	6.57	17.21	371.5	56.51	3.28
94	*Juniperus sabina* L.	Cupressaceae	AcS	PH	Sclerophyllous	3.6	0.75					0.87
95	*Jurinea humilis*(Desf.) DC.	Asteraceae	AcS	EHS	Leaf indument	2.84	0.45	17.83	23.03	443.6	24.88	1.08
96	*Linaria tristis* subsp. *tristis*	Veronicaceae	AcS	PH	-	8.4	0.5	30.1	11.94	435.6	14.47	1.21
97	*Marrubium supinum* L.	Lamiaceae	AcS	EHS	Seasonal dimorphism/leaf rolling	10.78	0.375	13.64	14.39	421.2	30.89	2.15
98	*Plantago subulata* L	Plantaginaceae	AcS	PH	-	3.4	0.34	24.5	91.82	435.2	17.76	0.19
99	*Prunus prostrata* Labill.	Rosaceae	AcS	DT	-	14.96	0.5	10.26	57.53	448.1	43.66	0.76
100	*Salvia lavandulifolia* Vahl	Lamiaceae	AcS	EHS	Leaf indument	19.82	0.5	31.32	31.19	430.9	13.76	0.44
101	*Satureja intricata* Lange	Lamiaceae	AcS	EHS	Seasonal dimorphism/leaf rolling	20	0.35	48.32	10.93	433.9	8.98	0.82
102	**Sideritis glacialis* Boiss.	Lamiaceae	AcS	EHS	Leaf indument	9.78	0.45	19.92	35.46	430.8	21.63	0.61
103	*Teucrium similatum* T.Navarro & Rosua	Lamiaceae	AcS	EHS	Seasonal dimorphism/leaf rolling	8.36	0.4	24.72	24.34	451.2	18.25	0.75
104	*Thymus mastichina* (L.) L.	Lamiaceae	AcS	EHS	Seasonal dimorphism/leaf rolling	4.59	0.36	34.23	38.49	438.5	12.81	0.33
105	*Vella spinosa* Boiss.	Brassicaceae	AcS	DLS	-	9.85	0.45	11.4	41.69	415.2	36.43	0.87
				MeanAcS	-	9.13	0.49	22.76	23.38	431.68	23.59	1.36
				Variance	-	19.48	0.02	99.26	344.79	814.51	156.89	0.76
				Std. Desviation	-	4.41	0.13	9.96	18.57	28.54	12.53	0.87
				Minimum	-	2.84	0.34	6.57	6.80	351.00	8.98	0.19
				Maximum	-	20.00	0.84	48.32	91.82	493.60	56.51	3.63
				Range	-	17.16	0.50	41.75	85.02	142.60	47.53	3.44
				n	27	27	27	27	27	27	27	27
106	*Acinos alpinus* subsp. *meridionalis (Nyman) P. W. Ball*	Lamiaceae	AjS	PH	-	12.28	0.5	30.65	12.14	431.6	14.08	1.16

107	*Arenaria tetraqueta* L.	Caryophyllaceae	AjS	EHS	None	11.88	0.57	31.29	8.44	433	13.84	1.64
108	*Astragalus nevadensis* Boiss.	Fabaceae	AjS	DHS	-	7.82	0.57	11.74	35.43	444.9	37.89	1.07
109	*Dianthus pungens* subsp. *brachyanthus* (Boiss.) B.Fern.Casas. G.López & M.Laínz	Caryophyllaceae	AjS	PH	-	12.43	0.5	20.99	16.55	439.8	20.95	1.27
110	*Digitalis purpurea* L. subsp. *purpurea*	Veronicaceae	AjS	PH	-	6.44	0.43	33.89	5.53	433.8	12.8	2.32
111	*Draba hispanica* Boiss.	Brassicaceae	AjS	PH	-	10.6	0.33					2.11
112	*Erigeron major* (Boiss.) Vierh.	Asteraceae	AjS	EHS	None	7.8	0.5	20.32	9.55	409.9	20.17	2.11
113	*Eryngium bourgatii* Gouan	Apiaceae	AjS	EHS	None	2.8	0.5	25.11	10.91	401	15.97	1.46
114	*Erysimum grandiflorum* Desf.	Brassicaceae	AjS	EHS	None	10.11	0.5	17.05	10.96	380.6	22.32	2.04
115	*Euphorbia nicaensis* All.	Euphorbiaceae	AjS	PH	-	15.15	0.45	12.04	14.47	435.1	36.14	2.5
116	*Helianthemun apenninum* subsp. *stoechadifolium (Brot.) Samp.*	Cistaceae	AjS	DHS	-	8.88	0.5	28.44	6.95	454.4	15.98	2.3
117	**Herniaria boissieri* Gay subsp. *boissieri*	Caryophyllaceae	AjS	EHS	Leaf indument	10.95	0.43	32.41		416.2	12.84	
118	*Hieracium amplexicaule* L.	Asteraceae	AjS	Evergreen half shub	None	7.85	0.45	20.2	19.46	447.3	22.14	1.14
119	*Hormathophylla spinosa* (L.) P. Küpfer	Brassicaceae	AjS	Evergreen half shub	Stems like leaf	10.26	0.45	16.54	13.96	410.4	24.81	1.78
120	*Jasione crispa* subsp. *amethystina* (Lag. & Rodr.) Tutin	Campanulaceae	AjS	Evergreen half shub	Leaf indument	18.34	0.45	17.66	12.98	427.6	24.21	1.86
121	*Juniperus sabina* L.	Cupressaceae	AjS	Evergreen tree	-	3.36	0.75	56.51	9.93	498.4	8.82	0.89
122	*Jurinea humilis* (Desf.) DC.	Asteraceae	AjS	Evergreen hulf shrub	Leaf indument	7.12	0.45	25.72	11.99	403.1	15.67	1.31
123	*Plantago subulata* L	Plantaginaceae	AjS	PH	-	8.85	0.43	26.8	11.24	306.9	11.45	1.02
124	**Sideritis glacialis* Boiss.	Lamiaceae	AjS	EHS	Leaf indument	12.15	0.38	14.25	17.7	443	31.08	1.76
125	**Teucrium aureum Schreb.* subsp. *angustifolium* (Willk.)Valdés Berm. & Sánchez Crespo	Lamiaceae	AjS	EHS	Seasonal dimorphism/leaf rolling	16.64	0.5	17.8	12.56	457.7	25.71	2.05
126	*Thymus granatensis* Boiss.	Lamiaceae	AjS	EHS	Seasonal dimorphism/leaf rolling	14.43	0.5	25.65	10.87	447	17.43	1.6
				MeanAjS	-	10.29	0.48	24.25	13.24	426.09	20.22	1.67
				Variance	-	15.61	0.01	103.37	40.79	1421.31	64.14	0.24
				Std. Desviation	-	3.95	0.08	10.17	6.39	37.70	8.01	0.49
				Minimum	-	2.80	0.33	11.74	5.53	306.90	8.82	0.89
				Maximum	-	18.34	0.75	56.51	35.43	498.40	37.89	2.50
				Range	-	15.54	0.42	44.77	29.90	191.50	29.07	1.61
				n	21	21	21	20	19	20	20	19
				Mean	-	8.28	0.53	25.56	19.13	432.05	19.81	1.28
				Std. Deviation	-	4.74	0.14	10.79	11.17	46.29	8.55	0.67
				Variance	-	22.45	0.02	116.47	124.84	2142.33	75.16	0.45
	All Mediterrean formations			Range	-	28.25	0.90	55.28	87.12	275.40	48.92	4.73
				Minimum	-	1.76	0.29	6.57	4.69	242.50	7.59	0.19
				Maximum	-	20.00	1.00	61.77	91.82	506.70	56.51	4.92
				n	126	126	126	125	123	124	124	123

Supplementary Material 1

Table S2: Leaf structural trait (SLA) and leaf nutrient traits (LNC and LPC) for species with drought of drought avoidance mechanisms according to Travlos and Chachalis (2008). Mean value and standard deviation (Mean ± SD), Coefficient of Variation (CV, defined as SD/Mean).

		SLA ($mm^2 mg^{-1}$)	LNC ($mg\ g^{-1}$)	LPC ($mg\ g^{-1}$)
Stems like leaf	Mean±SD	8.4± 6.1	25.83± 15	1.61± 1.27
	N	9	9	9
	CV (%)	78.37	58.07	78.88
Leaf indumentum	Mean±SD	8.22± 4.8	18.28± 6.32	1.15± 0.52
	N	21	21	19
	CV (%)	55.05	34.6	45.22
Seasonal dimorphis/ leaf rolling	Mean±SD	11.2± 4.31	17.03± 6.1	1.11± 0.56
	N	14	14	14
	CV (%)	42.27	35.82	50.45
