# Supplementary material for: Variations in Leaf Traits Modulate Plant Vegetative and Reproductive Phenological Sequencing Across Arid Mediterranean Shrublands
Source: Front Plant Sci. 2021 Aug 23;12:708367. doi: 10.3389/fpls.2021.708367 (PMC8420881; doi:10.3389/fpls.2021.708367)
Supplement: Supplementary file 2 [file Data_Sheet_2.PDF]

Supplementary Material 2

Supplementary material 2.-
Phenological calendars of the 126 perennial species. DVG = dolichoblast vegetative growth; FBF = flower bud formation; F = flowering; FS = fruit setting; PSI = Phenophase Sequence Index; NmDVG = number of months of vegetative growing; NmF = number of months of flowering; Nmo = number of month of overlapping between F and DVG.

SaMS: Semiarid mediterranean shrublands (Cabo de Gata)

Sp.		J	F	M	A	My	J	Jl	Au	S	O	N	D
Artemisia herba-alba	DVG												
	BVG												
	FBF												
	F												
	FS												
	APS	8	NmDVG		5	Nmo	2						
	PSI	0,5	NmF		3								
Asparagus acutifolius	DVG												
	BVG												
	FBF												
	F												
	FS												
	APS	12	NmDVG		10	Nmo	1						
	PSI	0,9	NmF		2								
Asparagus albus	DVG												
	BVG												
	FBF												
	F												
	FS												
	APS	12	NmDVG		10	Nmo	2						
	PSI	0,8	NmF		3								

SMS: Subdesert mediterranean shrublands (Tabernas)

Sp.		J	F	M	A	My	J	Jl	Au	S	O	N	D
Anabasis articulata	DVG												
	BVG												
	FBF												
	F												
	FS												
	APS	9	NmDVG		5	Nmo	1						
	PSI	0,82	NmF		4								
Anthyllis citisoides	DVG												
	BVG												
	FBF												
	F												
	FS												
	APS	12	NmDVG		8	Nmo	4						
	PSI	0,8	NmF		6								
Artemisia herba alba	DVG												
	BVG												
	FBF												
	F												
	FS												
	APS	8	NmDVG		6	Nmo	2						
	PSI	0,58	NmF		3								

Asparagus horridus	DVG					
	BVG					
	FBF					
	F					
	FS					
	APS	12	NmDVG	10	Nmo	3
	PSI	0,8	NmF	3		
Ballota hirsuta	DVG					
	BVG					
	FBF					
	F					
	FS					
	APS	12	NmDVG	5	Nmo	0
	PSI	0,7	NmF	5		
Chamaerops humilis	DVG					
	BVG					
	FBF					
	F					
	FS					
	APS	8	NmDVG	3	Nmo	0
	PSI	0,8	NmF	3		
Cistus albidus	DVG					
	BVG					
	FBF					
	F					
	FS					
	APS	5	NmDVG	3	Nmo	1
	PSI	0,5	NmF	2		
Asparagus horridus	DVG					
	BVG					
	FBF					
	F					
	FS					
	APS	12	NmDVG	9	Nmo	1
	PSI	0,79	NmF	3		
Carlina hispanica	DVG					
	BVG					
	FBF					
	F					
	FS					
	APS	9	NmDVG	0	Nmo	2
	PSI	0,64	NmF	5		
Cistus clusii	DVG					
	BVG					
	FBF					
	F					
	FS					
	APS	7	NmDVG	4	Nmo	0
	PSI	0,7	NmF	3		
Euzomodendron bourjeanu	DVG					
	BVG					
	FBF					
	F					
	FS					
	APS	12	NmDVG	7	Nmo	
	PSI	0,45	NmF	8		

Species						
Cistus salviifolius	DVG					
	BVG					
	FBF					
	F					
	FS					
	APS	5	NmDVG	4	Nmo	2
	PSI	0,7	NmF	3		
Coris monspeliensis	DVG					
	BVG					
	FBF					
	F					
	FS					
	APS	6	NmDVG	2	Nmo	2
	PSI	0,5	NmF	4		
Daphne gnidium	DVG					
	BVG					
	FBF					
	F					
	FS					
	APS	12	NmDVG	12	Nmo	10
	PSI	0,4	NmF	10		
Genista spartioides	DVG					
	BVG					
	FBF					
	F					
	FS					
	APS	6	NmDVG	4	Nmo	3
	PSI	0,5	NmF	4		
Frankenia corymbosa	DVG					
	BVG					
	FBF					
	F					
	FS					
	APS	12	NmDVG	5	Nmo	4
	PSI	0,61	NmF	9		
Hammada tamariscifolia	DVG					
	BVG					
	FBF					
	F					
	FS					
	APS	11	NmDVG	5	Nmo	0
	PSI	0,57	NmF	5		
Helianthemum almeriensis	DVG					
	BVG					
	FBF					
	F					
	FS					
	APS	8	NmDVG	4	Nmo	3
	PSI	0,41	NmF	6		
Launaea arborescens	DVG					
	BVG					
	FBF					
	F					
	FS					
	APS	12	NmDVG	5	Nmo	5
	PSI	0,41	NmF	12		

Lavatera mitima	DVG												
	BVG												
	FBF												
	F												
	FS												
	APS	6	NmDVG	5	Nmo	3							
	PSI	0,5	NmF	4									
Olea europaea	DVG												
	BVG												
	FBF												
	F												
	FS												
	APS	6	NmDVG	3	Nmo	1							
	PSI	0,6	NmF	3									
Phlomis lychnitis	DVG												
	BVG												
	FBF												
	F												
	FS												
	APS	6	NmDVG	3	Nmo	1							
	PSI	0,6	NmF	3									
Phlomis purpurea	DVG												
	BVG												
	FBF												
	F												
	FS												
	APS	6	NmDVG	4	Nmo	2							
	PSI	0,5	NmF	4									

Launaea lanifera	DVG												
	BVG												
	FBF												
	F												
	FS												
	APS	12	NmDVG	4	Nmo	1							
	PSI	0,41	NmF	3									
Lavandula multifida	DVG												
	BVG												
	FBF												
	F												
	FS												
	APS	12	NmDVG	8	Nmo	6							
	PSI	0,52	NmF	6									
Licium intricatum	DVG												
	BVG												
	FBF												
	F												
	FS												
	APS	12	NmDVG	5	Nmo	5							
	PSI	0,41	NmF	12									
Limonium insigne	DVG												
	BVG												
	FBF												
	F												
	FS												
	APS	11	NmDVG	9	Nmo	5							
	PSI	0,53	NmF	0									

Species						
Quercus coccifera	DVG					
	BVG					
	FBF					
	F					
	FS					
	APS	4	NmDVG	3	Nmo	2
	PSI	0,4	NmF	2		
Rhamnus lycioides	DVG					
	BVG					
	FBF					
	F					
	FS					
	APS	5	NmDVG	4	Nmo	2
	PSI	0,5	NmF	2		
Ruta chalepensis	DVG					
	BVG					
	FBF					
	F					
	FS					
	APS	4	NmDVG	3	Nmo	2
	PSI	0,4	NmF	2		
Teucrium charidemi	DVG					
	BVG					
	FBF					
	F					
	FS					
	APS	8	NmDVG	5	Nmo	0
	PSI	0,7	NmF	2		

Species						
Limonium majus	DVG					
	BVG					
	FBF					
	F					
	FS					
	APS	7	NmDVG	4	Nmo	0
	PSI	0,7	NmF	3		
Phagnalon saxatile	DVG					
	BVG					
	FBF					
	F					
	FS					
	APS	6	NmDVG	5	Nmo	2
	PSI	0,55	NmF	3		
Plantago lanceolata	DVG					
	BVG					
	FBF					
	F					
	FS					
	APS	8	NmDVG	5	Nmo	2
	PSI	0,54	NmF	4		
Thymus baeticus	DVG					
	BVG					
	FBF					
	F					
	FS					
	APS	9	NmDVG	7	Nmo	4
	PSI	0,6	NmF	4		

Teucrium hieronymi						
DVG						
BVG						
FBF						
F						
FS						
APS	7	NmDVG	4	Nmo	0	
PSI	0,8	NmF	2			

Teucrium lusitanicum						
DVG						
BVG						
FBF						
F						
FS						
APS	8	NmDVG	5	Nmo	3	
PSI	0,47	NmF	5			

Teucrium lusitanicum						
DVG						
BVG						
FBF						
F						
FS						
APS	6	NmDVG	4	Nmo	1	
PSI	0,6	NmF	3			

Thymelaea hirsuta						
DVG						
BVG						
FBF						
F						
FS						
APS	10	NmDVG	10	Nmo	7	
PSI	0,67	NmF	7			

Thymus hyemalis						
DVG						
BVG						
FBF						
F						
FS						
APS	6	NmDVG	4	Nmo	1	
PSI	0,6	NmF	2			

Retama sphaerocarpa						
DVG						
BVG						
FBF						
F						
FS						
APS	10	NmDVG	5	Nmo	1	
PSI	0,62	NmF	4			

Ulex canescens						
DVG						
BVG						
FBF						
F						
FS						
APS	4	NmDVG	3	Nmo	3	
PSI	0,4	NmF	3			

Salsola webbii						
DVG						
BVG						
FBF						
F						
FS						
APS	8	NmDVG	4	Nmo	2	
PSI	0,58	NmF	5			

Sp.		J	F	M	A	My	J	Jl	Au	S	O	N	D
Salsola papillosa	DVG												
	BVG												
	FBF												
	F												
	FS												
	APS	10	NmDVG		7	Nmo	2						
	PSI	0,69	NmF		4								
Salsola oppositifolia	DVG												
	BVG												
	FBF												
	F												
	FS												
	APS	11	NmDVG		4	Nmo	0						
	PSI	0,69	NmF		6								
Salsola genistoides	DVG												
	BVG												
	FBF												
	F												
	FS												
	APS	8	NmDVG		5	Nmo	2						
	PSI	0,54	NmF		4								

SAS: Subalpin shrublands (Sierra Nevada)

Sp.		J	F	M	A	My	J	Jl	Au	S	O	N	D
Alyssum serpyllifolium	DVG												
	BVG												
	FBF												
	F												
	FS												
	APS	6	NmDVG		3	Nmo	1						
	PSI	0,1	NmF		4								

AcS: Alpine cushion schrublands (Sierra Nevada)

Sp.		J	F	M	A	My	J	Jl	Au	S	O	N	D
Acinus alpinus meridionalis	DVG												
	BVG												
	FBF												
	F												
	FS												
	APS	5	NmDVG		4	Nmo	3						
	PSI	0,4	NmF		3								

Anthyllis vulneraria* subsp.*arundana	DVG					
	BVG					
	FBF					
	F					
	FS					
	APS	5	NmDVG	3	Nmo	1
	PSI	0,67	NmF	2		
Artemisa granatensis	DVG					
	BVG					
	FBF					
	F					
	FS					
	APS	7	NmDVG	4	Nmo	1
	PSI	0,6	NmF	3		
Berberis hispanica	DVG					
	BVG					
	FBF					
	F					
	FS					
	APS	5	NmDVG	2	Nmo	0
	PSI	0,67	NmF	2		
Bupleurum fruticenscens* subsp. *spinosum	DVG					
	BVG					
	FBF					
	F					
	FS					
	APS	8	NmDVG	3	Nmo	0
	PSI	0,64	NmF	4		

Alyssum serpilloides	DVG					
	BVG					
	FBF					
	F					
	FS					
	APS	6	NmDVG	3	Nmo	1
	PSI	0,7	NmF	4		
Anthyllis vulneraria* sbsp *arunda	DVG					
	BVG					
	FBF					
	F					
	FS					
	APS	5	NmDVG	3	Nmo	1
	PSI	0,84	NmF	2		
Artemisia granatensis	DVG					
	BVG					
	FBF					
	F					
	FS					
	APS	7	NmDVG	4	Nmo	1
	PSI	0,7	NmF	3		
Astragalus granatiensis	DVG					
	BVG					
	FBF					
	F					
	FS					
	APS	4	NmDVG	2	Nmo	1
	PSI	0,43	NmF	2		

Cotoneaster granatensis	DVG					
	BVG					
	FBF					
	F					
	FS					
	APS	5	NmDVG	2	Nmo	0
	PSI	0,67	NmF	2		
Crataegus monogyna	DVG					
	BVG					
	FBF					
	F					
	FS					
	APS	6	NmDVG	3	Nmo	2
	PSI	0,45	NmF	4		
Daphne oleoides	DVG					
	BVG					
	FBF					
	F					
	FS					
	APS	10	NmDVG	11	Nmo	3
	PSI	0,625	NmF	3		
Digitalis purpurea* subsp. *purpurea	DVG					
	BVG					
	FBF					
	F					
	FS					
	APS	7	NmDVG	5	Nmo	3
	PSI	0,4	NmF	4		
Erinacea anthyllis* subsp. *anthyllis	DVG					
	BVG					
	FBF					
	F					
	FS					
	APS	4	NmDVG	2	Nmo	2
	PSI	0,71	NmF	3		
Berberis hispanica	DVG					
	BVG					
	FBF					
	F					
	FS					
	APS	5	NmDVG	2	Nmo	0
	PSI	0,67	NmF	2		
Cerastium alpinum	DVG					
	BVG					
	FBF					
	F					
	FS					
	APS	4	NmDVG	3	Nmo	2
	PSI	0,56	NmF	3		
Chaenorrhinum glareosum	DVG					
	BVG					
	FBF					
	F					
	FS					
	APS		NmDVG	3	Nmo	3
	PSI	0,56	NmF	4		
Dianthus brachyanthus	DVG					
	BVG					
	FBF					
	F					
	FS					
	APS	5	NmDVG	3	Nmo	2
	PSI	0,45	NmF	3		
Draba hispanica	DVG					
	BVG					
	FBF					
	F					
	FS					
	APS	4	NmDVG	3	Nmo	3
	PSI	0,34	NmF	3		

Euphorbia nicaensis* subsp *nicaensis	DVG					
	BVG					
	FBF					
	F					
	FS					
	APS	5	NmDVG	3	Nmo	3
	PSI	0,44	NmF	4		
Helianthemun apenninum*subsp. *estevei	DVG					
	BVG					
	FBF					
	F					
	FS					
	APS	4	NmDVG	2	Nmo	1
	PSI	0,5	NmF	2		
Helichrysum stoechas	DVG					
	BVG					
	FBF					
	F					
	FS					
	APS	8	NmDVG	6	Nmo	
	PSI	0,39	NmF	6		
Helleborus foetidus	DVG					
	BVG					
	FBF					
	F					
	FS					
	APS	5	NmDVG	3	Nmo	2
	PSI	0,5	NmF	3		
Jurinea humilis	DVG					
	BVG					
	FBF					
	F					
	FS					
	APS	6	NmDVG	4	Nmo	2
	PSI	0,45	NmF	3		
Erinacea anthyllis* subsp *anthyllis	DVG					
	BVG					
	FBF					
	F					
	FS					
	APS	4	NmDVG	2	Nmo	
	PSI	0,43	NmF	3		
Euphorbia nicaensis	DVG					
	BVG					
	FBF					
	F					
	FS					
	APS	4	NmDVG	3	Nmo	3
	PSI	0,45	NmF	4		
Genista versicolor	DVG					
	BVG					
	FBF					
	F					
	FS					
	APS	4	NmDVG	3	Nmo	1
	PSI	0,5	NmF	2		
Helianthemun apenninum* subsp. *stoechdifolium	DVG					
	BVG					
	FBF					
	F					
	FS					
	APS	4	NmDVG	2	Nmo	1
	PSI	0,5	NmF	2		
Hormatophylla spinosa	DVG					
	BVG					
	FBF					
	F					
	FS					
	APS	6	NmDVG	3	Nmo	
	PSI	0,45	NmF	4		

Lavandula lanata	DVG					
	BVG					
	FBF					
	F					
	FS					
	APS	6	NmDVG	4	Nmo	2
	PSI	0,45	NmF	3		
Linum narbonense	DVG					
	BVG					
	FBF					
	F					
	FS					
	APS	4	NmDVG	3	Nmo	3
	PSI	0,36	NmF	4		
Lonicera arborea	DVG					
	BVG					
	FBF					
	F					
	FS					
	APS	6	NmDVG	4	Nmo	2
	PSI	0,45	NmF	3		
Onosma tricerosperma	DVG					
	BVG					
	FBF					
	F					
	FS					
	APS	7	NmDVG	5	Nmo	3
	PSI	0,43	NmF	4		
Phlomis crinita	DVG					
	BVG					
	FBF					
	F					
	FS					
	APS	6	NmDVG	4	Nmo	3
	PSI	0,42	NmF	4		

Juniperus sabina	DVG					
	BVG					
	FBF					
	F					
	FS					
	APS	3	NmDVG	2	Nmo	0
	PSI	0,75	NmF	1		
Jurinea humilis	DVG					
	BVG					
	FBF					
	F					
	FS					
	APS	6	NmDVG	4	Nmo	2
	PSI	0,45	NmF	3		
Linaria tristis* subsp. *tristis	DVG					
	BVG					
	FBF					
	F					
	FS					
	APS	3	NmDVG	2	Nmo	1
	PSI	0,5	NmF	1		
Marrubium supinum	DVG					
	BVG					
	FBF					
	F					
	FS					
	APS	4	NmDVG	3	Nmo	2
	PSI	0,375	NmF	3		
Plantago subulata	DVG					
	BVG					
	FBF					
	F					
	FS					
	APS	4	NmDVG	3	Nmo	3
	PSI	0,34	NmF	3		

Prunus mahaleb	DVG					
	BVG					
	FBF					
	F					
	FS					
	APS	4	NmDVG	2	Nmo	1
	PSI	0,42	NmF	2		
Prunus ramburii	DVG					
	BVG					
	FBF					
	F					
	FS					
	APS	4	NmDVG	2	Nmo	1
	PSI	0,5	NmF	2		
Quercus ilexsubsp. ballota	DVG					
	BVG					
	FBF					
	F					
	FS					
	SD					
	APS	5	NmDVG	0	Nmo	1
	PSI	0,5	NmF	0		
Rhamnus saxatilis	DVG					
	BVG					
	FBF					
	F					
	FS					
	APS	6	NmDVG	5	Nmo	2
	PSI	0,57	NmF	2		

Prunus prostata	DVG					
	BVG					
	FBF					
	F					
	FS					
	APS	6	NmDVG	4	Nmo	2
	PSI	0,5	NmF	3		
Salvia lavandufolia	DVG					
	BVG					
	FBF					
	F					
	FS					
	APS	4	NmDVG	3	Nmo	2
	PSI	0,5	NmF	2		
Satureja intrincata	DVG					
	BVG					
	FBF					
	F					
	FS					
	SD					
	APS	8	NmDVG	0	Nmo	5
	PSI	0,35	NmF	0		
Sideritis glacialis	DVG					
	BVG					
	FBF					
	F					
	FS					
	APS	5	NmDVG	3	Nmo	2
	PSI	0,45	NmF	4		

Rosa canina	DVG					
	BVG					
	FBF					
	F					
	FS					
	APS	5	NmDVG	5	Nmo	3
	PSI	0,5	NmF	3		
Salvia lavandulifolia	DVG					
	BVG					
	FBF					
	F					
	FS					
	APS	4	NmDVG	3	Nmo	2
	PSI	0,42	NmF	2		
Santolina rosmarinifolia	DVG					
	BVG					
	FBF					
	F					
	FS					
	APS	7	NmDVG	7	Nmo	5
	PSI	0,5	NmF	5		
Santolina chamaeryprissus	DVG					
	BVG					
	FBF					
	F					
	FS					
	APS	7	NmDVG	9	Nmo	5
	PSI	0,41	NmF	5		

Teucrium similatum	DVG					
	BVG					
	FBF					
	F					
	FS					
	APS	5	NmDVG	3	Nmo	3
	PSI	0,4	NmF	4		
Thymus mastichina	DVG					
	BVG					
	FBF					
	F					
	FS					
	APS	5	NmDVG	3	Nmo	3
	PSI	0,36	NmF	4		
Vella spinosa	DVG					
	BVG					
	FBF					
	F					
	FS					
	APS	6	NmDVG	3	Nmo	2
	PSI	0,45	NmF	4		

Teucrium similatum	DVG												
	BVG												
	FBF												
	F												
	FS												
	APS	5	NmDVG		3	Nmo	2						
	PSI	0,44	NmF		3								
Thymus mastichina	DVG												
	BVG												
	FBF												
	F												
	FS												
	APS	5	NmDVG		3	Nmo	3						
	PSI	0,36	NmF		4								
Ulex parviflorus	DVG												
	BVG												
	FBF												
	F												
	FS												
	APS	5	NmDVG		3	Nmo	3						
	PSI	0,36	NmF		4								
**AjS: Alpine Juniper prostrate shrublands (Sierra Nevada)**													
Sp.		J	F	M	A	My	J	Jl	Au	S	O	N	D
Acinos alpinus* subsp. *meridionales	DVG												
	BVG												
	FBF												
	F												
	FS												
	APS	5	NmDVG		4	Nmo	3						
	PSI	0,5	NmF		3								

Arenaria tetraqueta	DVG					
	BVG					
	FBF					
	F					
	FS					
	APS	4	NmDVG	3	Nmo	2
	PSI	0,57	NmF	2		
Astragalus nevadensis	DVG					
	BVG					
	FBF					
	F					
	FS					
	APS	4	NmDVG	2	Nmo	1
	PSI	0,57	NmF	2		
Dianthus brachyanthus	DVG					
	BVG					
	FBF					
	F					
	FS					
	APS	5	NmDVG	3	Nmo	2
	PSI	0,5	NmF	3		
Digitalis purpurea	DVG					
	BVG					
	FBF					
	F					
	FS					
	APS	7	NmDVG	5	Nmo	3
	PSI	0,43	NmF	4		
Draba hispanica	DVG					
	BVG					
	FBF					
	F					
	FS					
	APS	4	NmDVG	3	Nmo	3
	PSI	0,33	NmF	3		

Erigeron major	DVG												
	BVG												
	FBF												
	F												
	FS												
	APS	4	NmDVG		2	Nmo	1						
	PSI	0,5	NmF		2								
Eryngium bourgati	DVG												
	BVG												
	FBF												
	F												
	FS												
	APS	4	NmDVG		2	Nmo	1						
	PSI	0,5	NmF		2								
Erysimum grandiflorum	DVG												
	BVG												
	FBF												
	F												
	FS												
	APS	4	NmDVG		2	Nmo	1						
	PSI	0,5	NmF		2								
Euphorbia nicaensis	DVG												
	BVG												
	FBF												
	F												
	FS												
	APS	5	NmDVG		3	Nmo	3						
	PSI	0,45	NmF		4								
Helianthemun apenninum* subsp. *stoechdifolium	DVG												
	BVG												
	FBF												
	F												
	FS												
	APS	4	NmDVG		2	Nmo	1						
	PSI	0,5	NmF		2								

Herniaria boissieri* subsp *boissieri	DVG												
	BVG												
	FBF												
	F												
	FS												
	APS	4	NmDVG		3	Nmo	2						
	PSI	0,43	NmF		2								
Hieracium amplexicaule	DVG												
	BVG												
	FBF												
	F												
	FS												
	APS	5	NmDVG		3	Nmo	2						
	PSI	0,45	NmF		3								
Hormatophylla spinosa	DVG												
	BVG												
	FBF												
	F												
	FS												
	APS	6	NmDVG		3	Nmo	2						
	PSI	0,45	NmF		4								
Jasione crispa* subsp *amethystina	DVG												
	BVG												
	FBF												
	F												
	FS												
	APS	4	NmDVG		3	Nmo	2						
	PSI	0,45	NmF		3								
Juniperus sabina	DVG												
	BVG												
	FBF												
	F												
	FS												
	APS	3	NmDVG		2	Nmo	0						
	PSI	0,75	NmF		1								

Jurinea humilis	DVG											
	BVG											
	FBF											
	F											
	FS											
	APS	6	NmDVG	4	Nmo	2						
	PSI	0,45	NmF	3								
Plantago subulata	DVG											
	BVG											
	FBF											
	F											
	FS											
	APS	3	NmDVG	3	Nmo	2						
	PSI	0,43	NmF	2								
Sideritis glacilis	DVG											
	BVG											
	FBF											
	F											
	FS											
	APS	5	NmDVG	3	Nmo	3						
	PSI	0,38	NmF	5								
Teucrium aureum* subsp *angustifolium	DVG											
	BVG											
	FBF											
	F											
	FS											
	APS	4	NmDVG	2	Nmo	2						
	PSI	0,5	NmF	3								
Thymus granatensis	DVG											
	BVG											
	FBF											
	F											
	FS											
	APS	4	NmDVG	3	Nmo	2						
	PSI	0,5	NmF	3								
