# Supplementary material for: Variations in Leaf Traits Modulate Plant Vegetative and Reproductive Phenological Sequencing Across Arid Mediterranean Shrublands
Source: Front Plant Sci. 2021 Aug 23;12:708367. doi: 10.3389/fpls.2021.708367 (PMC8420881; doi:10.3389/fpls.2021.708367)
Supplement: Supplementary file 3 [file Data_Sheet_3.PDF]

Supplementary Material 3

Table S1.- Results from univariate ANOVAs testing differences between leaf functional traits and formations. For each analysis, *F*-value is shown. n.s. (not significant). Specific leaf area (SLA), LCC, LNC and LPC (leaf carbon, leaf nitrogen and leaf phosphorous content respectively), leaf C:N:P stoichiometry, and Phenological index (PSI). N= 22 in Semiarid Mediterranean Shrubland (SaMS); N = 26 in Subdesert Mediterranean Shrubland (SMS); N = 30 in Subalpine Shrubland (SAS); N= 27 in Alpine Cushion Schrubland (AcS); N= 21 in Alpine Juniper prostrate Shrubland (AjS). Degrees of freedom (df) = 4.

	F	Sig.
SLA (mm^2 mg^{-1})	2.63	0.04
LNC (mg g^{-1})	3.17	0.02
LCC (mg g¯1)	6.89	0.00
LPC (mg g^{-1})	5.19	0.00
N:P	4.11	0.00
C:N	4.93	0.00
PSI index	3.00	0.02

Table S2.- Results from univariate ANOVAs testing differences between leaf functional traits and functional groups. For each analysis, *F*-value is shown. n.s. (not significant). Specific leaf area (SLA), LCC, LNC and LPC (leaf carbon, leaf nitrogen and leaf phosphorous content respectively), leaf C:N:P stoichiometry, and Phenological index (PSI). N= 27 in Perennial Herbs (PH); N = 13 in Deciduous Half Shrubs (DHS); N= 5 in Deciduous Large Shrubs (DLS); N= 48 in Evergreen Half Shrubs (EHS); N= 15 in Evergreen Large Shrubs (ELS); N= 5 in Deciduous Trees (DT); N= 5 in Evergreen Trees (ET) and N = 8 in Succulents (SC); df = 7.

	F	Sig.
SLA (mm^2 mg^{-1})	3.64	0.00
LNC (mg g^{-1})	2.393	0.03
LCC (mg g¯1)	5.375	0.00
LPC (mg g^{-1})	0.672 n. s	0.70
N:P	2.85	0.01
C:N	0.59 n. s	0.77
PSI index	1.67 n. s	0.12
