# Supplementary material for: Variations in Leaf Traits Modulate Plant Vegetative and Reproductive Phenological Sequencing Across Arid Mediterranean Shrublands
Source: Front Plant Sci. 2021 Aug 23;12:708367. doi: 10.3389/fpls.2021.708367 (PMC8420881; doi:10.3389/fpls.2021.708367)
Supplement: Supplementary file 4 [file Data_Sheet_4.PDF]

Supplementary Material 4

Table S1. Pearson correlation coefficients (*r*) and significance among leaf functional traits for all studied species.

* Correlation significant at the 0.05 level (2-tailed). ** Correlation significant at the 0.01 level (2-tailed). Specific leaf area (SLA). LCC, LNC and LPC (leaf carbon. leaf nitrogen and leaf phosphorous content respectively), leaf C:N:P stoichiometry and Phenological index (PSI).

All species/Leaf traits		SLA	LNC	LPC	LCC	C:N	N:P	PSI index
**SLA**	**Pearson correlation**	1.00	0.28**	0.37**	-0.05	-0.27**	-0.02*	-0.17
	**Sig. (bilateral)**		0.00	0	0.59	0.00	0.02	0.06
**LNC**	**Pearson correlation**	0.28**	1.00	0.60**	-0.18*	-0.97**	0.08	0.05
	**Sig. (bilateral)**	0.00		0.00	0.05	0.00	0.37	0.57
**LPC**	**Pearson correlation**	0.38**	0.60**	1.00	-0.19*	0.60**	-0.75**	0.03
	**Sig. (bilateral)**		0.00		0.04	0.00	0.00	0.75
**LCC**	**Pearson correlation**	-0.08	0.08	0.42**	1.00	-0.19	-0.18*	-0.05
	**Sig. (bilateral)**	0.36	0.41	0.00		0.04	0.05	0.59
**N:P**	**Pearson correlation**	-0.22*	0.08	-0.75**	0.08	-0.06	1.00	-0.02
	**Sig. (bilateral)**	0.02	0.37	0.00	0.41	0.54		0.82
**C:N**	**Pearson correlation**	-0.27**	-0.98**	-0.60**	0.42**	1.00	-0.06	-0.07
	**Sig. (bilateral)**	0.00	0.00	0.00	0.00		0.54	0.44
**N:P**	**Pearson correlation**	-0.22*	0.08	-0.75**	0.08	-0.06	1.00	-0.02
	**Sig. (bilateral)**	0.02	0.37	0.00	0.41	0.54		0.82
**PSI index**	**Pearson correlation**	0.17	0.05	0.03	-0.08	-0.07	-0.02	1.00
	**Sig. (bilateral)**	0.06	0.57	0.75	0.36	0.44	0.82	

Table S2. Pearson correlation coefficients (*r*) and significance among leaf functional traits at formation level. * Correlation significant at the 0.05 level (2-tailed). ** Correlation significant at the 0.01 level (2-tailed). Specific leaf area (SLA). LCC. LNC and LPC (leaf carbon. leaf nitrogen and leaf phosphorous content respectively). leaf C:N:P stoichiometry and Phenological index (PSI).

Formations/leaf traits		SLA	LNC	LPC	LCC	C:N	N:P	PSI index
**Subdesert mediterranean shrublands (SMS)**								
**SLA**	**Pearson correlation**	1.00	0.30	0.54**	0.02	-0.26	-0.485*	-0.58**
	**Sig. (bilateral)**		0.12	0.00	0.90	0.20	0.01	0.00
**LNC**	**Pearson correlation**	0.30	1.00	0.71**	-0.11	-0.93**	-0.08	-0.24
	**Sig. (bilateral)**	0.13		0.00	0.59	0.00	0.69	0.25
**LPC**	**Pearson correlation**	0.54**	0.71**	1.00	-0.01	-0.63**	-0.77**	-0.50*
	**Sig. (bilateral)**	0.00	0.00		0.95	0.00	0.00	0.01
**LCC**	**Pearson correlation**	0.03	-0.11	-0.01	1.00	0.48*	-0.08	-0.06
	**Sig. (bilateral)**	0.90	0.59	0.95		0.01	0.69	0.78
**C:N**	**Pearson correlation**	-0.26	-0.93**	-0.63**	0.477*	1.00	0.04	0.19
	**Sig. (bilateral)**	0.20	0.00	0.00	0.01		0.84	0.36
**N:P**	**Pearson correlation**	-0.49*	-0.08	-0.77**	-0.08	0.04	1.00	0.480*
	**Sig. (bilateral)**	0.01	0.69	0.00	0.69	0.84		0.01
**PSI index**	**Pearson correlation**	-0.56**	-0.24	-0.50*	-0.06	0.19	0.48*	1.00
	**Sig. (bilateral)**	0.00	0.25	0.01	0.78	0.36	0.01	
**Semiarid mediterranean shrublands (SaMS)**								
**SLA**	**Pearson correlation**	1.00	0.42	0.55**	-0.36	-0.45*	-0.50*	0.14
	**Sig. (bilateral)**		0.05	0.01	0.10	0.03	0.02	0.54
**LNC**	**Pearson correlation**	0.42	1.00	0.78**	-0.21	-.99**	-0.44*	0.02
	**Sig. (bilateral)**	0.05		0.00	0.34	0.00	0.04	0.94
**LPC**	**Pearson correlation**	0.55**	0.78**	1.00	-0.11	-0.76**	-0.91**	0.17

	**Sig. (bilateral)**	0.01	0.00		0.64	0.00	0.00	0.45
**LCC**	**Pearson correlation**	-0.36	-0.21	-0.11	1.00	0.35	0.01	0.21
	**Sig. (bilateral)**	0.10	0.34	0.64		0.11	0.96	0.36
**C:N**	**Pearson correlation**	-0.45*	-0.99**	-0.76**	0.35	1.00	0.42	0.02
	**Sig. (bilateral)**	0.03	0.00	0.00	0.11		0.05	0.95
**N:P**	**Pearson correlation**	-0.50*	-0.44*	-0.92**	0.01	0.42	1.00	-0.23
	**Sig. (bilateral)**	0.02	0.04	0.00	0.96	0.05		0.30
**PSI index**	**Pearson correlation**	0.14	0.01	0.17	0.21	0.02	-0.23	1.00
	**Sig. (bilateral)**	0.54	0.94	0.45	0.36	0.95	0.30	
**Subalpin shrublands (SAS)**								
**SLA**	**Pearson correlation**	1.00	0.33	0.12	-0.30	-0.37*	0.20	0.26
	**Sig. (bilateral)**		0.07	0.54	0.11	0.04	0.29	0.16
**LNC**	**Pearson correlation**	0.33	1.00	0.75**	-0.25	-0.98**	-0.10	0.51**
	**Sig. (bilateral)**	0.07		0.00	0.18	0.00	0.62	0.00
**LPC**	**Pearson correlation**	0.12	0.75**	1.00	-0.04	-0.67**	-0.73**	0.35
	**Sig. (bilateral)**	0.54	0.00		0.82	0.00	0.00	0.06
**LCC**	**Pearson correlation**	-0.30	-0.25	-0.04	1.00	0.45*	-0.23	-0.31
	**Sig. (bilateral)**	0.11	0.18	0.82		0.01	0.23	0.09
**C:N**	**Pearson correlation**	-0.37*	-0.99**	-0.70**	0.45*	1.00	0.03	-0.53**
	**Sig. (bilateral)**	0.04	0.00	0.00	0.01		0.86	0.00
**N:P**	**Pearson correlation**	0.20	-0.10	-0.73**	-0.23	0.03	1.00	0.07
	**Sig. (bilateral)**	0.29	0.62	0.00	0.23	0.86		0.69
**PSI index**	**Pearson correlation**	0.26	0.51**	0.35	-0.31	-0.53**	0.08	1.00
	**Sig. (bilateral)**	0.16	0.00	0.06	0.09	0.00	0.69	
**Alpine cushion schrublands (AcS)**								
**SLA**	**Pearson correlation**	1.00	0.22	0.31*	-0.10	-0.24	-0.11	-0.09

	**Sig. (bilateral)**		0.13	0.04	0.50	0.11	0.46	0.53
**LNC**	**Pearson correlation**	0.22	1.00	0.39**	0.02	-0.98**	0.36*	-0.01
	**Sig. (bilateral)**	0.13		0.01	0.91	0.00	0.02	0.99
**LPC**	**Pearson correlation**	0.31*	0.39**	1.00	-0.23	-0.42**	-0.72**	0.23
	**Sig. (bilateral)**	0.04	0.01		0.13	0.00	0.00	0.13
**LCC**	**Pearson correlation**	-0.10	0.02	-0.23	1.00	0.16	0.24	0.04
	**Sig. (bilateral)**	0.50	0.91	0.13		0.27	0.12	0.77
**C:N**	**Pearson correlation**	-0.24	-0.98**	-0.42**	0.16	1.00	-0.31*	0.01
	**Sig. (bilateral)**	0.11	0.00	0.00	0.27		0.04	0.94
**N:P**	**Pearson correlation**	-0.11	0.36*	-0.72**	0.24	-0.31*	1.00	-0.34*
	**Sig. (bilateral)**	0.46	0.02	0.00	0.12	0.04		0.03
**PSI index**	**Pearson correlation**	-0.09	0.00	0.23	0.04	0.01	-0.36*	1.00
	**Sig. (bilateral)**	0.53	0.99	0.13	0.77	0.94	0.03	
**Alpine *Juniper* prostrate shrublands (AjS)**								
**SLA**	**Pearson correlation**	1.00	0.47*	0.40	0.01	-0.47*	0.18	-0.38
	**Sig. (bilateral)**		0.04	0.08	0.95	0.04	0.46	0.09
**LNC**	**Pearson correlation**	0.47*	1.00	0.37	0.15	-0.97**	0.69**	-0.31
	**Sig. (bilateral)**	0.04		0.12	0.52	0.00	0.00	0.18
**LPC**	**Pearson correlation**	0.40	0.37	1.00	0.08	-0.35	-0.42	-0.45*
	**Sig. (bilateral)**	0.08	0.12		0.73	0.15	0.07	0.05
**LCC**	**Pearson correlation**	0.01	0.15	0.08	1.00	0.09	0.08	0.43
	**Sig. (bilateral)**	0.95	0.52	0.73		0.70	0.75	0.06
**C:N**	**Pearson correlation**	-0.47*	-0.97**	-0.35	0.09	1.00	-0.67**	0.42
	**Sig. (bilateral)**	0.04	0.00	0.15	0.70		0.00	0.07
**N:P**	**Pearson correlation**	0.18	0.69**	-0.42	0.08	-0.67**	1.00	-0.05

	**Sig. (bilateral)**	0.46	0.00	0.07	0.75	0.00		0.85
**PSI index**	**Pearson correlation**	-0.38	-0.31	-0.45*	0.43	0.42	-0.05	1.00
	**Sig. (bilateral)**	0.09	0.18	0.05	0.06	0.07	0.85	

Table S3. Pearson correlation coefficients (*r*) and significance among leaf functional traits at functional group level.

* Correlation significant at the 0.05 level (2-tailed). ** Correlation significant at the 0.01 level (2-tailed). Specific leaf area (SLA). LCC. LNC and LPC (leaf carbon. leaf nitrogen and leaf phosphorous content respectively). leaf C:N:P stoichiometry and Phenological index (PSI).

**Functional groups/traits**		**SLA**	**LNC**	**LPC**	**LCC**	**C:N**	**N:P**	**PSI index**
**SC (succulents)**								
**SLA**	**Pearson correlation**	1.00	0.64	0.84**	-0.50	-0.72*	-0.77*	-0.34
	**Sig. (bilateral)**		0.09	0.01	0.21	0.04	0.03	0.41
**LNC**	**Pearson correlation**	0.64	1.00	0.76*	-0.29	-0.89**	-0.44	0.07
	**Sig. (bilateral)**	0.09		0.03	0.49	0.00	0.28	0.87
**LPC**	**Pearson correlation**	0.84**	0.76*	1.00	-0.26	-0.70	-0.92**	-0.32
	**Sig. (bilateral)**	0.01	0.03		0.53	0.05	0.00	0.43
**LCC**	**Pearson correlation**	-0.50	-0.29	-0.26	1.00	0.69	0.19	-0.16
	**Sig. (bilateral)**	0.21	0.49	0.53		0.06	0.65	0.70
**C:N**	**Pearson correlation**	-0.72*	-0.89**	-0.70	0.69	1.00	0.42	-0.13
	**Sig. (bilateral)**	0.04	0.00	0.05	0.06		0.30	0.76
**N:P**	**Pearson correlation**	-0.77*	-0.44	-0.92**	0.19	0.42	1.00	0.50
	**Sig. (bilateral)**	0.03	0.28	0.00	0.65	0.30		0.21
**PSI index**	**Pearson correlation**	-0.34	0.07	-0.33	-0.16	-0.13	0.50	1.00
	**Sig. (bilateral)**	0.41	0.87	0.43	0.70	0.76	0.21	
**PH (perennial herbs)**								

**SLA**	**Pearson correlation**	1.00	0.41*	0.58**	-0.26	-0.47*	-0.39	-0.23
	**Sig. (bilateral)**		0.04	0.00	0.20	0.02	0.05	0.27
**LNC**	**Pearson correlation**	0.41*	1.00	0.44*	0.01	-0.96**	0.11	-0.10
	**Sig. (bilateral)**	0.04		0.02	0.96	0.00	0.59	0.64
**LPC**	**Pearson correlation**	0.58**	0.44*	1.00	-0.24	-0.49*	-0.84**	-0.19
	**Sig. (bilateral)**	0.00	0.02		0.24	0.01	0.00	0.36
**LCC**	**Pearson correlation**	-0.26	0.01	-0.24	1.00	0.27	0.27	0.03
	**Sig. (bilateral)**	0.20	0.96	0.24		0.19	0.18	0.88
**C:N**	**Pearson correlation**	-0.47*	-0.96**	-0.49*	0.27	1.00	-0.03	0.10
	**Sig. (bilateral)**	0.02	0.00	0.01	0.19		0.88	0.63
**N:P**	**Pearson correlation**	-0.39	0.11	-0.84**	0.27	-0.03	1.00	0.09
	**Sig. (bilateral)**	0.05	0.59	0.00	0.18	0.88		0.67
**PSI index**	**Pearson correlation**	-0.23	-0.09	-0.19	0.03	0.10	0.09	1.00
	**Sig. (bilateral)**	0.265	0.643	0.361	.883	0.626	0.669	
**ELS (evergreen large shrubs)**								
**SLA**	**Pearson correlation**	1.00	0.65*	0.61*	-0.55*	-0.68*	-0.29	0.02
	**Sig. (bilateral)**		0.01	0.02	0.04	0.01	0.36	0.98
**LNC**	**Pearson correlation**	0.65*	1.00	0.86**	-0.52	-0.98**	-0.30	0.12
	**Sig. (bilateral)**	0.01		0.00	0.09	0.00	0.34	0.69
**LPC**	**Pearson correlation**	0.61*	0.86**	1.00	-0.57*	-0.87**	-0.74**	0.31
	**Sig. (bilateral)**	0.02	0.00		0.06	0.00	0.01	0.31
**LCC**	**Pearson correlation**	-0.55*	-0.52	-0.57*	1.00	0.63*	0.38	0.03
	**Sig. (bilateral)**	0.04	0.09	0.06		0.03	0.22	0.34
**C:N**	**Pearson correlation**	-0.67*	-0.99**	-0.87**	0.63*	1.00	0.33	-0.06
	**Sig. (bilateral)**	0.01	0.00	0.00	0.03		0.28	0.83

**N:P**	**Pearson correlation**	-0.29	-0.30	-0.74**	0.38	0.33	1.00	-0.43
	**Sig. (bilateral)**	0.36	0.34	0.01	0.22	0.28		0.16
**PSI index**	**Pearson correlation**	0.02	0.12	0.31	0.30	-0.06	-0.43	1.00
	**Sig. (bilateral)**	0.98	0.69	0.31	0.34	0.83	0.16	
**EHS (evergreen half shrubs)**								
**SLA**	**Pearson correlation**	1.00	0.02	0.10	-0.17	-0.06	-0.09	-0.01
	**Sig. (bilateral)**		0.90	0.50	0.22	0.71	0.50	0.89
**LNC**	**Pearson correlation**	0.02	1.00	0.69**	-0.29*	-0.98**	-0.06	0.14
	**Sig. (bilateral)**	0.90		0.00	0.04	0.00	0.69	0.31
**LPC**	**Pearson correlation**	0.10	0.69**	1.00	-0.21	-0.68**	-0.76**	0.18
	**Sig. (bilateral)**	0.50	0.00		0.13	0.00	0.00	0.21
**LCC**	**Pearson correlation**	-0.17	-0.29*	-0.21	1.00	0.48**	0.01	0.01
	**Sig. (bilateral)**	0.22	0.04	0.13		0.00	0.93	0.95
**C:N**	**Pearson correlation**	-0.06	-0.98**	-0.68**	0.48**	1.00	0.06	-0.13
	**Sig. (bilateral)**	0.71	0.00	0.00	0.00		0.70	0.36
**N:P**	**Pearson correlation**	-0.09	-0.06	-0.76**	0.01	0.06	1.00	-0.12
	**Sig. (bilateral)**	0.50	0.69	0.00	0.93	0.70		0.42
**PSI index**	**Pearson correlation**	-0.01	0.14	0.18	0.01	-0.13	-0.12	1.00
	**Sig. (bilateral)**	0.89	0.31	0.21	0.95	0.36	0.42	
**DT (deciduous trees)**								
**SLA**	**Pearson correlation**	1.00	0.50	-0.45	-0.55	-0.57	0.60	0.20
	**Sig. (bilateral)**		0.75	0.42	0.13	0.22	0.11	0.402
**LNC**	**Pearson correlation**	0.52	1.00	-0.33	0.12	-0.99**	0.87*	-0.14
	**Sig. (bilateral)**	0.75		0.27	0.70	0.00	0.04	0.89
**LPC**	**Pearson correlation**	-0.45	-0.33	1.00	0.35	0.37	-0.77	0.01

	**Sig. (bilateral)**	0.41	0.27		0.61	0.78	0.33	0.95
**LCC**	**Pearson correlation**	-0.55	0.12	0.35	1.00	-0.03	-0.15	0.28
	**Sig. (bilateral)**	0.13	0.70	0.61		0.06	0.04	0.48
**C:N**	**Pearson correlation**	-0.57	-0.1**	0.37	-0.03	1.00	-0.87*	0.17
	**Sig. (bilateral)**	0.41	0.00	0.35	0.99		0.89	0.987
**N:P**	**Pearson correlation**	0.60	0.86*	-0.77	-0.12	-0.87*	1.00	-0.10
	**Sig. (bilateral)**	0.97	0.03	0.42	0.13	0.05		0.40
**PSI index**	**Pearson correlation**	0.20	-0.14	0.01	0.28	0.17	-0.10	1.00
	**Sig. (bilateral)**	0.40	0.89	0.95	0.84	0.69	0.89	
**DHS (deciduous half shrubs)**								
**SLA**	**Pearson correlation**	1.00	0.64*	0.79**	0.41	-0.57*	-0.31	-0.05
	**Sig. (bilateral)**		0.02	0.00	0.18	0.05	0.33	0.89
**LNC**	**Pearson correlation**	0.64*	1.00	0.51	0.16	-0.98**	0.31	0.05
	**Sig. (bilateral)**	0.02		0.09	0.61	0.00	0.33	0.88
**LPC**	**Pearson correlation**	0.79**	0.51	1.00	0.26	-0.47	-0.68*	-0.09
	**Sig. (bilateral)**	0.00	0.09		0.42	0.12	0.02	0.76
**LCC**	**Pearson correlation**	0.41	0.16	0.26	1.00	0.02	-0.14	-0.31
	**Sig. (bilateral)**	0.18	0.61	0.42		0.95	0.66	0.33
**C:N**	**Pearson correlation**	-0.57*	-0.98**	-0.47	0.02	1.00	-0.34	-0.11
	**Sig. (bilateral)**	0.05	0.00	0.12	0.95		0.28	0.74
**N:P**	**Pearson correlation**	-0.31	0.31	-0.66*	-0.14	-0.34	1.00	0.16
	**Sig. (bilateral)**	0.33	0.33	0.02	0.66	0.28		0.63
**PSI index**	**Pearson correlation**	-0.05	0.05	-0.09	-0.31	-0.11	0.16	1.00
	**Sig. (bilateral)**	0.89	0.88	0.76	0.33	0.74	0.63	
**DLS (deciduous large shrubs)**								
**SLA**	**Pearson correlation**	1.00	0.59	0.66	0.18	-0.58	-0.37	0.78

	**Sig. (bilateral)**		0.30	0.22	0.77	0.31	0.54	0.12
**LNC**	**Pearson correlation**	0.59	1.00	0.81	0.03	-0.99**	-0.08	0.35
	**Sig. (bilateral)**	0.30		0.10	0.96	0.00	0.90	0.56
**LPC**	**Pearson correlation**	0.66	0.81	1.00	0.43	-0.78	-0.65	0.78
	**Sig. (bilateral)**	0.22	0.10		0.47	0.12	0.24	0.12
**LCC**	**Pearson correlation**	0.18	0.03	0.43	1.00	0.05	-0.69	0.59
	**Sig. (bilateral)**	0.77	0.96	0.47		0.94	0.20	0.30
**C:N**	**Pearson correlation**	-0.57	-0.99**	-0.78	0.05	1.00	0.03	-0.31
	**Sig. (bilateral)**	0.31	0.00	0.12	0.94		0.97	0.61
**N:P**	**Pearson correlation**	-0.37	-0.08	-0.65	-0.69	0.03	1.00	-0.87
	**Sig. (bilateral)**	0.54	0.90	0.24	0.20	0.97		0.06
**PSI index**	**Pearson correlation**	0.78	0.35	0.78	0.59	-0.31	-0.87	1.00
	**Sig. (bilateral)**	0.12	0.56	0.12	0.30	0.61	0.06	
