# Supplementary material for: Variations in Leaf Traits Modulate Plant Vegetative and Reproductive Phenological Sequencing Across Arid Mediterranean Shrublands
Source: Front Plant Sci. 2021 Aug 23;12:708367. doi: 10.3389/fpls.2021.708367 (PMC8420881; doi:10.3389/fpls.2021.708367)
Supplement: Supplementary file 5 [file Data_Sheet_5.PDF]

Supplementary Material 5

Table S1.- Comparison of the leaf structural traits (SLA), leaf nutrients (LCC, LNC, and LPC), and leaf C:N:P stoichiometry between our study and other existing studies. Arid Mediterranean Shrubland categories: Semiarid Mediterranean Shrubland (SaMS), Subdesert Mediterranean Shrubland (SMS); Subalpine Shrubland (SAS); Alpine Cushion Schrubland (AcS), and Alpine *Juniper prostrate* Shrubland (AjS). Functional groups categories: Evergreen Trees (ET); Deciduous Trees (DT), Evergreen Large Shrubs (ELS); Evergreen Half Shrubs (EHS); Deciduous Large Shrubs (DLS); Deciduous Half Shrubs (DHS); Succulents (SC) and Perennial Herbs (PH). SD: Standard deviation.

**Data source**	**Ecosystems**	**C (mg g^{-1})**	**N (mg g^{-1})**	**P(mg g^{-1})**	**C:N (mass)**	**C:P (mass)**	**N:P (mass)**	**SLA (mm^2 mg^{-1})**
				**Our study**				
Mean±SD		432.1±43.0	19,8±8.7	1.3±0.8	25.6±10.41	—	18.9±11	8.3±4
n		126	126	126	126	—	126	126
				**Reich & Oleksyn (2004)**				
Mean±SD		461.6±72.2	20.1±8.7	1.77±1.1	23.8±17.3	300.9±236.8	13.8±9.5	—
n		76	1251	932	62	43	894	—
				**Dominguez et al., 2012**				**LMA (g m^{-2})**
Mean±SD		503±31.1	15.6±5.1	1±0.5	—	—	—	103.6±28
n		17	17	17	—	—	—	17
				**Fyllas et al., 2009**				
Mean±SD		471.5	20.7	0.9	—	—	—	—

n		289	293	290	—	—	—	—
**Fyllas et al., 2020**								**LMA (g m^{-2})**
Mean±SD	Needleleaf Evergreens	—	1.16±0.3	0.09±0.0	—	—	—	189.8±44.2
n	Broadleaf Deciduous	—	2.3±0.3	0.2±0.0	—	—	—	61.3±15.6
	Broadleaf Evergreens	—	1.3±0.1	0.1±0.0	—	—	—	132.6±19
**Villar & Merino (2001)**								
Mean±SD	Xeric mediterranean forest	—	22.04±7.7	—	—	—	—	5.1±1.8
n	Mesic Mediterranean Forest	—	15.7±5.2	—	—	—	—	7.7±3.6
	17	10	—	—	—	—	—	
Mean±SD	—	0.19	0.97	—	—	—	—	2.08±0.3
n								
**Hernández et al., 2011**								
Mean±SD		456.6±1.6	10.7±0.6	—	—	—	—	6.6±0.4
n								
**Güsewell (2004)**								
**Mediterranean (Greece)**								
Mean±SD	Evergreen woody	—	10.3	0.62	—	—	16.6	—

n	Deciduous woody	—	23.0	1.53	—	—	15.1	—
Mean±SD	Herbaceous (mainly forbs)	—	17.5	1.25	—	—	14.0	—
**Mediterranean (Australia)**								
Mean±SD	Woody	—	9.5	0.7	—	—	13.6	—
Mean±SD	Herbaceous	—	10.6	0.9	—	—	11.8	—
**World-wide**								
Mean±SD	Evergreen woody	—	13.7	1.0	—	—	13.4	—
Mean±SD	Deciduous woody	—	22.2	1.6	—	—	13.9	—
Mean±SD	Forbs	—	22.2	1.9	—	—	11.9	—
Mean±SD	Graminoids	—	16.0	1	—	—	17.8	—

* Logarithm
